# Potential preventive effects of selected traditional Chinese medicine as adjuvant therapy on hypertensive heart disease progression by replenishing qi and activating blood circulation: a systematic review and meta-analysis of clinical trials

**DOI:** 10.3389/fphar.2025.1506234

**Published:** 2025-10-02

**Authors:** Jiaqi Hui, Ya Wang, Fengqin Xu, Junnan Zhao

**Affiliations:** ^1^ Institute of Geriatrics, Xiyuan Hospital, China Academy of Chinese Medical Sciences, Beijing, China; ^2^ Laboratory of Combining Diseases and Evidence to Prevent Vascular Aging, National Administration of Traditional Chinese Medicine, Beijing, China; ^3^ Dongzhimen Hospital, Beijing University of Chinese Medicine, Beijing, China

**Keywords:** traditional Chinese medicine, therapy of replenishing qi and activating bloodcirculation, hypertensive heart disease, randomized controlled trial, systematic review, meta-analysis

## Abstract

**Objective:**

Hypertension remained an important public health problem with high morbidity and mortality and was emerging as a risk factor for future heart failure. The transition from hypertension to hypertensive heart disease (HHD) and heart failure grew progressively with time. Traditional Chinese medicine (TCM) has a history of several thousand years, where selected TCM for replenishing qi and activating blood circulation provides an alternative treatment for HHD.

**Methods:**

An extensive literature search was conducted across eight electronic databases from their inception until 8 September 2023, to evaluate the potential preventive effects of selected TCM as an adjuvant therapy on the progression of HHD. The outcome measures included blood pressure and indicators of cardiac structure and function under cardiac ultrasound. The mean difference (MD) and 95% confidence interval (CI) were used to determine continuous outcomes. Risk ratio (RR) with 95% confidence interval (CI) was used to determine dichotomous outcomes. The information about the overall certainty of the evidence from studies was presented according to specific outcomes using the Grading of Recommendations Assessment, Development and Evaluation (GRADE) Guideline Development Tool (GDT) online software.

**Results:**

Twenty-one randomized controlled trials (RCTs) involving 2, 055 participants were included. Meta-analyses favored integrated Chinese botanical drugs and Western medicine on blood pressure, New York Heart Association classification, left ventricular ejection fraction, transmitral peak early diastolic velocity/peak late diastolic velocity ratio, left ventricular internal diameters, left ventricular mass index, interventricular septum thickness in diastole, and B-type natriuretic peptide compared with Western medicine alone. Results on cardiac output should be interpreted with caution due to sample size limitations. No severe adverse events were identified. Most of the Chinese botanical drugs originated from classical TCM formulas. The dosage form of Chinese botanical drugs was oral. *Salvia miltiorrhiza* Bunge (Danshen), *Oreocome striata* (DC.) Pimenov & Kljuykov (Chuanxiong), *Pueraria montana* var. *lobata* (Willd.) Maesen & S.M.Almeida ex Sanjappa & Predeep (Gegen), *Astragalus mongholicus* Bunge (Huangqi), and *Typha angustifolia* L. (Puhuang) were the top 5 Chinese botanical drugs, which might be associated with replenishing qi and activating blood circulation.

**Conclusion:**

Selected TCM had the potential to be effective as an adjuvant therapy for alleviating adverse left ventricular remodeling and improving cardiac function after HHD, and therapy of replenishing qi and activating blood circulation may serve as a potential reference for treatment. To better assess Chinese botanical drugs’ preventative effects, more long-term, high-quality RCTs are still necessary.

**Systematic Review Registration:**

https://www.crd.york.ac.uk/PROSPERO/#myprospero, identifier CRD42022346030.

## Graphical Abstract

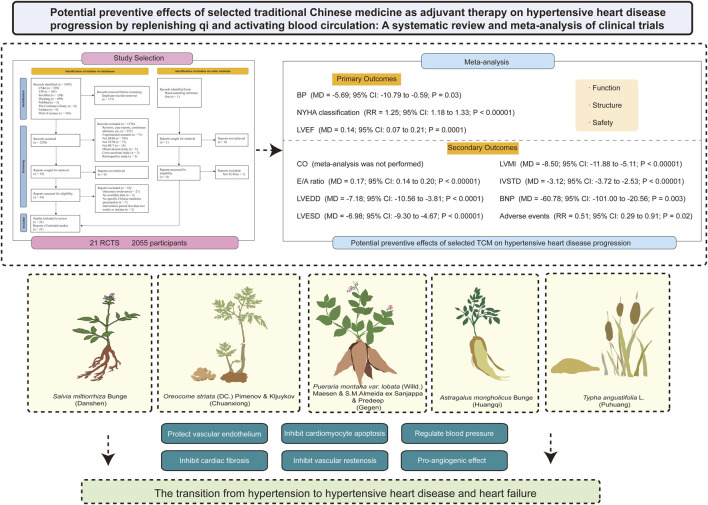

## 1 Introduction

Hypertensive Heart Disease (HHD), defined as symptomatic heart failure (HF) due to direct and long-term exposure to hypertension, was one of the most serious effects with its nonfatal burden derived from the model of HF (Escaned and Lerman, 2020; Roth et al., 2017). In a 20-year follow-up of 5, 143 participants from the Framingham Heart Study cohort, of all newly diagnosed HF patients, 91% had hypertension before developing HF (Levy et al., 1996). A recent study brought attention to the importance of early-stage hypertension as a significant aetiological risk factor for the development of early HF (Tromp et al., 2021) that compared the age variation in incident HF risk variables in the general population. In young participants, hypertension was associated with a threefold increase in the chance of developing HF later on. In contrast to a 1.4-fold risk in elderly participants (>65 years), in young participants (<55 years), hypertension was associated with a three-fold increase in the chance of developing HF later on (Bayés-Genís and Díez, 2022). Thus, elevated blood pressure (BP) was an essential risk factor for HF and, at the same time, a preventable cause (Virani et al., 2020; GBD, 2017 Risk Factor Collaborators, 2018). However, the early identification of patients with hypertension at risk of developing HF remains a challenge for clinicians (Escaned and Lerman, 2020). Long-term hypertension could cause hemodynamic stress that eventually changes the structure and metabolism of the myocardium. This could lead to cardiac remodeling, which showed up as HF and left ventricular (LV) dysfunction, and irregularities in myocardial perfusion and cardiac rhythm (Drazner, 2011; González et al., 2018; Bayés-Genís and Díez, 2022). Based on the clinical effects and pathophysiology of hypertension in the heart, HHD was divided into four ascending categories, including Degree Ⅰ (Isolated LV diastolic dysfunction with no LV hypertrophy (LVH)), Degree Ⅱ (LV diastolic dysfunction with concentric LVH), Degree Ⅲ (Clinical HF (dyspnea and pulmonary edema with preserved ejection fraction)), Degree Ⅳ (Dilated cardiomyopathy with HF and reduced ejection fraction) (Messerli et al., 2017; Iriarte et al., 1993). Therefore, HHD could be clinically asymptomatic or present with palpitations, chest tightness, dyspnea, biventricular failure, and sudden death (Dai et al., 2021). The results in the diagnosis of HHD largely rely on echocardiography and electrocardiogram (Dai et al., 2021; Devereux et al., 1993). Transthoracic echocardiography is the gold standard for noninvasive evaluation of cardiac structure and function. This provides a basis for assessing changes in cardiac structure during the shift from hypertension to HHD and HF. Previous studies suggested the adverse effects of hypertension on the heart (Ekhteiari Salmas et al., 2018; Salmas et al., 2017; Selamoglu Talas, 2014). Propolis is a resinous product collected by honeybees from various plant sources, which decreases tyrosine hydroxylase activity of the heart in nitric oxide synthase-inhibited hypertensive rats and thereby may modulate the synthesis of catecholamine and BP (Gogebakan et al., 2012). Antihypertensive medications, by definition, reduce BP, and when used as initial therapy, the majority of antihypertensive medications slowed the progression from hypertension to HF. However, examining the research on antihypertensive medications showed that not all of them have the same ability to prevent HF, for example, once-daily, low-dose hydrochlorothiazide was not recommended (Messerli et al., 2017). Thus, it was urgently needed to search for supplementary and alternative medical treatments for more effective control of HHD.

The investigation of traditional Chinese medicine (TCM) has the potential to lay an invaluable foundation for the development of new therapeutics. Multiple traditional botanical drugs and their metabolites, which are well-known for their proven excellent pharmacological effects, have long been utilized to treat different diseases, specifically cardiovascular disorders (Tavolinejad et al., 2019; Yousefsani et al., 2021). With the increasingly clinical application of selected TCM for replenishing qi and activating blood circulation in the therapy and prevention of cardiovascular diseases (Liu and Huang, 2016), therapy of replenishing qi and activating blood circulation has become an important role as a supplement and alternative treatment in clinical practice. Up till now, some randomized controlled trials (RCTs) have reported the effect of TCM on patients with HHD. The impact of selected TCM as an adjuvant therapy for the advancement of HHD disease was examined in this meta-analysis.

## 2 Materials and methods

The Preferred Reporting Items for Systematic Reviews and Meta-Analyses (PRISMA) reporting guideline recommendations were adhered to in this systematic review (Page et al., 2021), and Supplementary Table S1 contained the PRISMA checklist. The systematic review protocol was registered with International Prospective Register of Systematic Reviews (PROSPERO) (CRD42022346030) on 23 July 2022, prior to the initiation of study screening. Since this study involved a meta-analysis of data that had already been published, Ethics and Institutional Review Board approval was not necessary.

### 2.1 Search strategy

Eight electronic databases were systematically searched, including PubMed, the Cochrane Library, Embase, Web of Science, Wanfang Database, China National Knowledge Infrastructure (CNKI), Chinese Scientific Journal Database (Chinese VIP Information), and Chinese Biomedical Database (SinoMed) from inception to 8 September 2023, with no language or publication restrictions applied. Grey literature searches included Web of Science Conference Proceedings Citation Index-Science (CPCI-S), ClinicalTrials.gov (www.clinicaltrials.gov/), the World Health Organization International Clinical Trials Registry Platform (WHO ICTRP) (www.who.int/ictrp/en/), and International Traditional Medicine Clinical Trial Registry (ITMCTR) (itmctr.ccebtcm.org.cn/) using key terms and scanning reference lists of relevant reviews. ‘Medicine, Chinese Traditional’ was used as the Medical Subject Heading and matched with corresponding free words for enhancing accuracy. Given the discrepancy between databases, the keywords were adjusted flexibly for ‘hypertensive heart disease’ and ‘hypertensive cardiovascular disease.’ Search strategies were adapted to the specific syntax and controlled vocabulary of each database. Finally, all retrieval expressions were formed by logically connecting AND or OR. For example, the PubMed Database was searched as follows:

#1 (hypertensive heart disease [Title/Abstract]) OR (hypertensive cardiovascular disease [Title/Abstract])

#2 “Medicine, Chinese Traditional” [Mesh]

#3 (((((((((Traditional Chinese Medicine [Title/Abstract]) OR (Chung I Hsueh [Title/Abstract])) OR (Hsueh, Chung I [Title/Abstract])) OR (Traditional Medicine, Chinese [Title/Abstract])) OR (Zhong Yi Xue [Title/Abstract])) OR (Chinese Traditional Medicine [Title/Abstract])) OR (Chinese Medicine, Traditional [Title/Abstract])) OR (Drugs, Chinese Herbal [Title/Abstract])) OR (Complementary Therapies [Title/Abstract])) OR (Alternative Medicine [Title/Abstract])

#4 #2 OR #3.

#5 #1 AND #4.

The full search strategy is shown in Supplementary Table S2.

### 2.2 Inclusion and exclusion criteria

Study eligibility criteria were defined using the PICOS (Participants, Intervention, Comparators, Outcomes, Study design) approach. The inclusion criteria were as follows: (1) patients received a diagnosis of HHD without restrictions on gender, age, ethnicity, or disease stage; (2) patients in the TCM group were treated with Chinese botanical drugs based on those in the control group. Chinese botanical drugs was administered orally at least two-week-long treatment interventions, including Chinese patent medicine, single botanical drug, or TCM prescription; (3) the control group received conventional pharmacological interventions (Western medicine (WM); (4) the primary outcomes included BP (including systolic blood pressure (SBP) and diastolic blood pressure (DBP)), New York Heart Association (NYHA) classification, and left ventricular ejection fraction (LVEF); the secondary outcomes included cardiac output (CO), transmitral peak early diastolic velocity (E)/peak late diastolic velocity (A) ratio (E/A ratio), left ventricular internal diameters (including left ventricular end-diastolic diameter (LVEDD) and left ventricular end-systolic diameter (LVESD)), left ventricular mass index (LVMI), interventricular septum thickness in diastole (IVSTD), B-type natriuretic peptide (BNP), and adverse events; (5) the included RCTs were reported in completed paper article. To prevent duplication, we kept the most current publication or the most informative single article where the same population was published in multiple publications.

The exclusion criteria were as follows: (1) interventions included nonoral Chinese botanical drugs or appropriate TCM techniques; (2) it was not reported which botanical drugs were included in the TCM prescription containing multiple botanical drugs, nor the dosage of each type of botanical drug used; (3) it was not reported the administration method of the Chinese patent medicine, including the frequency of administration and the single oral dosage; (4) no relevant outcomes or no available data were reported; (5) the intervention period was less than 2 weeks or not reported; (6) the types of studies were reviews, case reports, retrospective studies, etc.

### 2.3 Study selection and data extraction

All identified indexed records were downloaded into EndNote X9, and duplicates were removed. After that, two review authors (J. Hui and Y. Wang) separately went through the titles and abstracts and evaluated the full-text publications to look for studies that might be included. Following PRISMA criteria, a flow chart contained the records of the research selection within the systematic review. Until data extractors achieved convergence and agreement, a standard data extraction form was created and tested. Independently, two review authors retrieved study characteristics and outcome data, including characteristics of the author, year, patients (e.g., age, gender, sample size), medication details for the experimental and control group, and outcome indicators. When there were several endpoint indicators in the literature, the longest one was chosen. If any clarification or further information was required, the corresponding authors of the original studies were contacted. Conflicts in data extraction were handled by the third review author (J. Zhao).

### 2.4 Methodological quality assessment

Two review authors (J. Hui and Y. Wang) independently assessed the risk of bias of all included RCTs using the Cochrane tool for assessing the risk of bias (Higgins et al., 2011). We resolved differences by discussion or by appeal to a third review author (J. Zhao). Following the recommendations of the Cochrane Handbook, the methodological quality was evaluated using seven domains: incomplete outcome data (attrition bias), selective reporting (reporting bias), blinding of participants and personnel (performance bias), random sequence generation (selection bias), allocation concealment (selection bias), and other bias. Three categories were used to classify each domain: low risk of bias, high risk of bias, and uncertain risk of bias. The original authors were contacted to verify and authenticate the randomization and allocation concealment procedures. If the original authors did not communicate, disagreements were settled by debate.

### 2.5 Data synthesis and statistical analysis

Statistical analysis was carried out using Review Manager 5.3 software (Cochrane Collaboration, Denmark). The mean difference (MD) or standardized mean difference (SMD) with 95% confidence interval (CI) was used to integrate continuous outcomes. SMD was used when different scales were used across studies. Dichotomous outcomes were calculated as risk ratio (RR) with 95% CI. We used the Chi^2^ (χ^2^) test and I^2^ statistic to quantify heterogeneity across included studies, where an I^2^ of 25% or less was regarded as low heterogeneity, an I^2^ of 26%–50% was regarded as moderate heterogeneity, and an I^2^ of over 50% was regarded as substantial heterogeneity (Higgins et al., 2003). When there was little to no heterogeneity (I^2^ ≤ 50%), a fixed-effects model was employed; when there was significant heterogeneity (I^2^ ˃ 50%), a random-effects model was used. All two-tailed P < 0.05 were considered statistically significant. Numerous participant- or intervention-related characteristics might be connected to heterogeneity among studies. If substantial heterogeneity was detected, we would perform subgroup and sensitivity analyses to investigate possible sources of heterogeneity between studies. Subsequently, sensitivity analyses were carried out by repeating the meta-analysis and removing each study one at a time to assess the robustness and dependability of the findings.

### 2.6 Quality of evidence

We summarized the quality of the evidence using the Grading of Recommendations Assessment, Development and Evaluation (GRADE) Guideline Development Tool (GDT) (www.gradepro.org). A final quality rating of high, moderate, low, or very poor was assigned to the evidence based on factors such as research design, risk of bias, inconsistency, indirectness, imprecision, and other factors.

### 2.7 Publication bias

The assessment of publication bias may be limited if there are insufficient studies for the outcomes. Using Stata 12.0 software, a visual assessment of the funnel plot was used to assess publication bias. Asymmetry indicated publication bias. In the meantime, funnel plot asymmetry was statistically demonstrated using Egger’s test. Publication bias does not exist if P > 0.05, and *vice versa*. The study’s analysis was adjusted for the impact of publication bias using the Duval and Tweedie trim-and-fill method.

## 3 Results

### 3.1 Study selection

**FIGURE 1:**
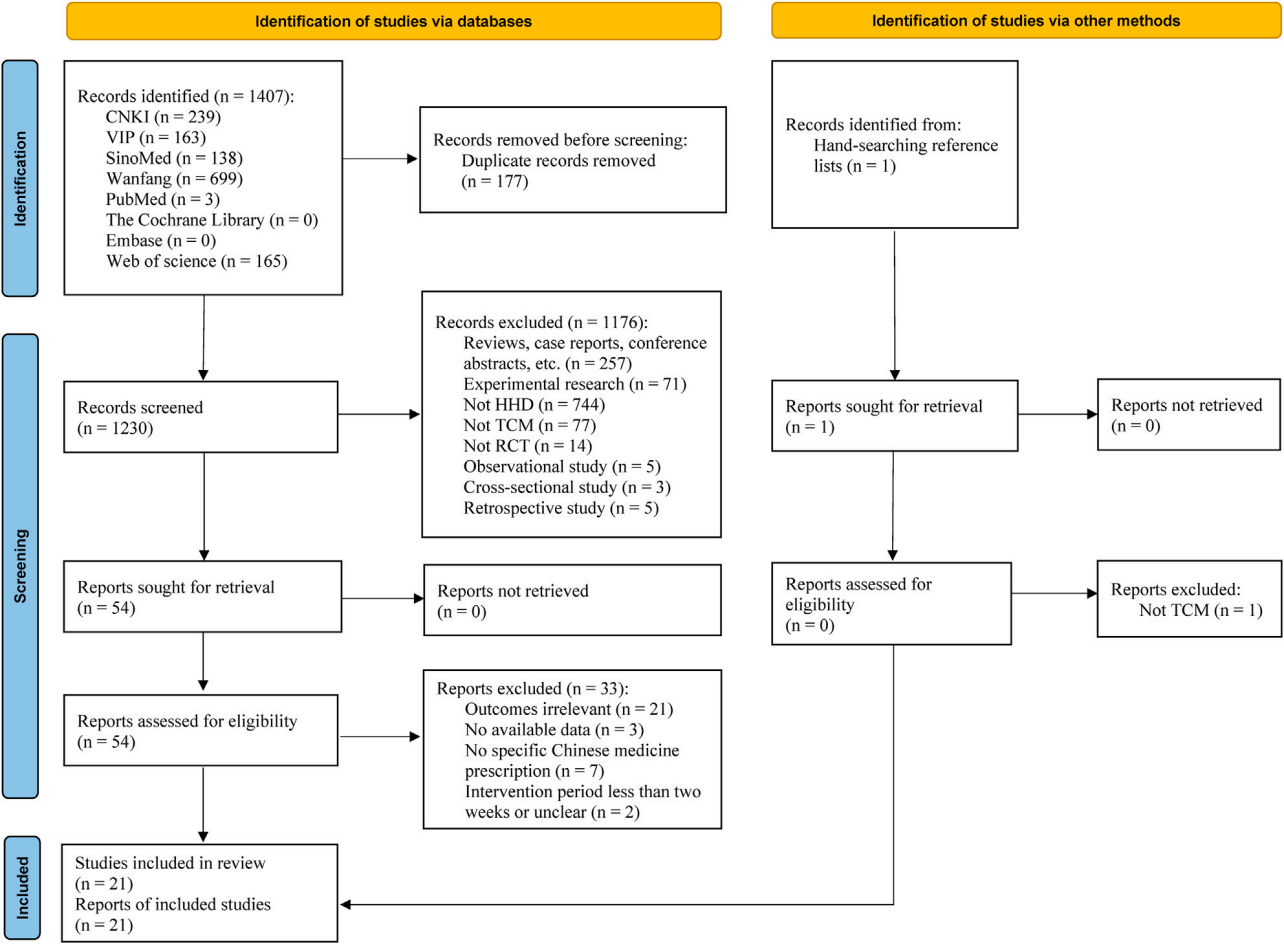
Flow diagram of the study selection process. Abbreviation: CNKI: China National Knowledge Infrastructure; HHD: hypertensive heart disease; RCT: randomized controlled trial; TCM: traditional Chinese medicine; VIP: Chinese Scientific Journal Database (Chinese VIP Information); SinoMed: Chinese Biomedical Database.

The literature searching process and research identification are summarized in Figure 1. A total of 1, 408 records were identified; 1, 407 from the database search approach, and one more study was found by looking through the reviews’ recognized references. In brief, for the 1,407 records via databases, following the initial database search and the removal of duplicate records, 1, 230 records were found. 1, 176 records were removed after additional title and abstract screening, mostly due to their lack of relevance to the study’s objectives. 33 of the 54 records that were subjected to a full-text review were eliminated because 21 of them contained only outcomes that were not relevant, three did not provide available outcome data, seven did not report the specific TCM prescription, and the other 2 records an unclear intervention periods or were of less than 2 weeks. Lastly, 21 studies were included in the review. Additionally, one study that was found by manually scanning the reference list was eliminated because it had no bearing on the goal of the investigation.

### 3.2 Characteristics of included studies

The baseline characteristics of included 21 RCTs were summarized in Table 1 (Cai, 2016; Cui, 2022; Du, 2016; Hou et al., 2015; Hu, 2012; Jin and Wu, 2006; Li et al., 2019; Liu, 2012; Peng, 2015; Song, 2011; Song, 2019; Tan, 2019; Tao, 2021; Wang and Wang, 2012; Wang et al., 2012; Wang and Sun, 2018; Yang and Zhou, 2002; Yuan, 2018; Zhang, 2013; Zhao, 2022; Zhu et al., 2019). The studies were published between 2002 and 2022. The demographics and clinical features of the population that was part of the meta-analysis were homogenous. A total of 2,055 individuals were involved in the research, with 1, 177 males and 878 females. The ages of the participants varied from 18 to 83 years. In 15 studies, the course of disease was reported, but not in the others. With 1, 041 patients in the CHM group and 1, 014 patients in the control group, the sample sizes of the included trials varied from 48 to 210. The control group received only WM treatment, while all CHM groups received oral CHM plus WM. The included studies’ treatment durations varied from 25 days to 12 months. For outcome measures, 6 (6/21, 28.6%) RCTs (Cai, 2016; Peng, 2015; Tan, 2019; Wang and Sun, 2018; Yang and Zhou, 2002; Yuan, 2018) reported BP, including SBP and DBP; 11 (11/21, 52.4%) RCTs (Cai, 2016; Hu, 2012; Li et al., 2019; Peng, 2015; Song, 2011; Tan, 2019; Wang and Wang, 2012; Wang et al., 2012; Wang and Sun, 2018; Yang and Zhou, 2002; Zhao, 2022) reported NYHA classification; 13 (13/21, 61.9%) RCTs (Cui, 2022; Du, 2016; Jin and Wu, 2006; Li et al., 2019; Song, 2019; Tan, 2019; Tao, 2021; Wang et al., 2012; Wang and Sun, 2018; Yang and Zhou, 2002; Yuan, 2018; Zhang, 2013; Zhu et al., 2019) reported LVEF; 2 (2/21, 9.5%) RCTs (Du, 2016; Tao, 2021) reported CO; 3 (3/21, 14.3%) RCTs (Cai, 2016; Li et al., 2019; Wang and Wang, 2012) reported E/A ratio; 9 (9/21, 42.9%) RCTs (Hou et al., 2015; Liu, 2012; Song, 2019; Tan, 2019; Wang and Sun, 2018; Yuan, 2018; Zhang, 2013; Zhao, 2022; Zhu et al., 2019) reported LVEDD; 4 (4/21, 19.0%) RCTs (Tan, 2019; Wang and Sun, 2018; Zhao, 2022; Zhu et al., 2019) reported LVESD; 2 (2/21, 9.5%) RCTs (Hou et al., 2015; Liu, 2012) reported LVMI; 3 (3/21, 14.3%) RCTs (Li et al., 2019; Tan, 2019; Wang and Sun, 2018) reported IVSTD; 3 (3/21, 14.3%) RCTs (Song, 2011; Wang and Sun, 2018; Zhao, 2022) reported BNP; and 8 (8/21, 38.1%) RCTs (Cai, 2016; Cui, 2022; Hu, 2012; Peng, 2015; Song, 2011; Wang et al., 2012; Wang and Sun, 2018; Yang and Zhou, 2002) reported adverse events.

**TABLE 1:** Characteristics of included studies.

References	Age (mean ± SD, year)		Gender (male/female)	Sample size (T/C)	Course of disease (mean ± SD, year)		Intervention (T/C)	Duration	Outcome indicator
	T	C			T	C			
Cai (2016)	56–76 (63.1 ± 2.2)	55–78 (63.5 ± 2.1)	41/34	38/37	NR	NR	TCM + WM/WM	2 months	①②⑤⑪
Cui (2022)	52–82 (67.13 ± 14.87)	49–83 (66.23 ± 16.77)	61/61	61/61	4–16 (10.33 ± 5.67)	5–15 (10.24 ± 4.76)	TCM + WM/WM	3 months	③⑪
Du (2016)	30–81 (55.3 ± 7.2)	31–78 (52.3 ± 6.8)	105/105	105/105	1–15 (7.8 ± 3.9)	1–16 (7.9 ± 3.8)	TCM + WM/WM	90 days	③④
Hou et al. (2015)	40–80 (59.44 ± 11.27)	39–78 (58.89 ± 11.39)	60/38	49/49	NR	NR	TCM + WM/WM	1 month	⑥⑧
Hu (2012)	34–75 (55 ± 6.7)	33–77 (55 ± 7.6)	95/47	72/70	1–21 (14 ± 2.1)	1–23 (14 ± 2.3)	TCM + WM/WM	25 days	②⑪
Jin and Wu (2006)	53–78	55–78	36/28	34/30	NR	NR	TCM + WM/WM	6 months	②③
Li et al. (2019)	18–70 (62.13 ± 8.24)	18–70 (61.02 ± 9.02)	71/41	56/56	6.88 ± 1.26	7.21 ± 1.37	TCM + WM/WM	2 months	②③⑤⑨
Liu (2012)	39–79 (56.3 ± 6.4)	36–78 (56.2 ± 6.5)	121/75	98/98	NR	NR	TCM + WM/WM	12 weeks	⑥⑧
Peng (2015)	32–76 (61.8 ± 10.2)	32–74 (60.2 ± 11.4)	44/52	48/48	NR	NR	TCM + WM/WM	12 months	①②⑪
Song (2011)	48–65		50/35	42/43	NR	NR	TCM + WM/WM	4 weeks	②⑩⑪
Song (2019)	41–72 (62.6 ± 7.3)	40–72 (62.5 ± 7.2)	57/39	48/48	1–6 (2.4 ± 0.5)	1–6 (2.3 ± 0.6)	TCM + WM/WM	4 weeks	③⑥
Tan (2019)	53–78 (65.5 ± 2.2)	51–76 (63.5 ± 1.8)	46/34	40/40	1–8 (4.5 ± 2.2)	1–6 (3.5 ± 1.5)	TCM + WM/WM	3 months	①②③⑥⑦⑨
Tao (2021)	31–74 (55.98 ± 8.7)	30–75 (56.23 ± 7.6)	33/27	30/30	1–16 (8.45 ± 0.94)	1–15 (8.49 ± 0.93)	TCM + WM/WM	2 months	③④
Wang and Wang (2012)	59–78	62–82	58/48	53/53	2–6	2–6	TCM + WM/WM	2 months	②⑤
Wang et al. (2012)	42–56	40–58	33/27	30/30	2–7		TCM + WM/WM	24 weeks	②③⑪
Wang and Sun (2018)	42–76 (68.59 ± 8.13)	40–81 (65.06 ± 7.28)	47/43	45/45	1–5 (2.19 ± 0.46)	1–6 (2.38 ± 0.61)	TCM + WM/WM	2 months	①②③⑥⑦⑨⑩⑪
Yang and Zhou (2002)	32–72 (58.88 ± 9.64)	34–73 (56.98 ± 8.52)	54/31	56/29	0.5–30 (4.00 ± 5.64)	0.67–32 (4.10 ± 4.82)	TCM + WM/WM	2 months	①③⑪
Yuan (2018)	46–77 (61.4 ± 8.5)	48–79 (63.0 ± 9.3)	59/33	46/46	1–5 (3.2 ± 1.7)	1–6 (3.5 ± 1.5)	TCM + WM/WM	2 months	①③⑥
Zhang (2013)	41–73 (63.9 ± 7.2)		41/27	30/38	1–5 (1.5 ± 0.4)		TCM + WM/WM	4 weeks	③⑥
Zhao (2022)	44–76 (63.58 ± 7.92)	43–75 (63.45 ± 7.89)	37/33	35/35	1–6.5 (2.40 ± 0.49)	1.5–6 (2.32 ± 0.52)	TCM + WM/WM	2 months	②⑥⑦⑩
Zhu et al. (2019)	45–75 (58.2 ± 6.5)	45–75 (56.5 ± 6.0)	28/20	25/23	21.5 ± 2.5	18.5 ± 1.8	TCM + WM/WM	6 months	③⑥⑦

C: the control group; NR: not reported; T: the TCM group; TCM: traditional Chinese medicine; WM: western medicine; ①: Blood pressure (including systolic and diastolic blood pressure); ②: New York Heart Association classification; ③: left ventricular ejection fraction; ④: cardiac output; ⑤: E/A ratio; ⑥: left ventricular end-diastolic diameter; ⑦: left ventricular end-systolic diameter; ⑧: left ventricular mass index; ⑨: interventricular septum thickness in diastole; ⑩: B-type natriuretic peptide; ⑪: adverse events.

### 3.3 Risk of bias

**FIGURE 2:**
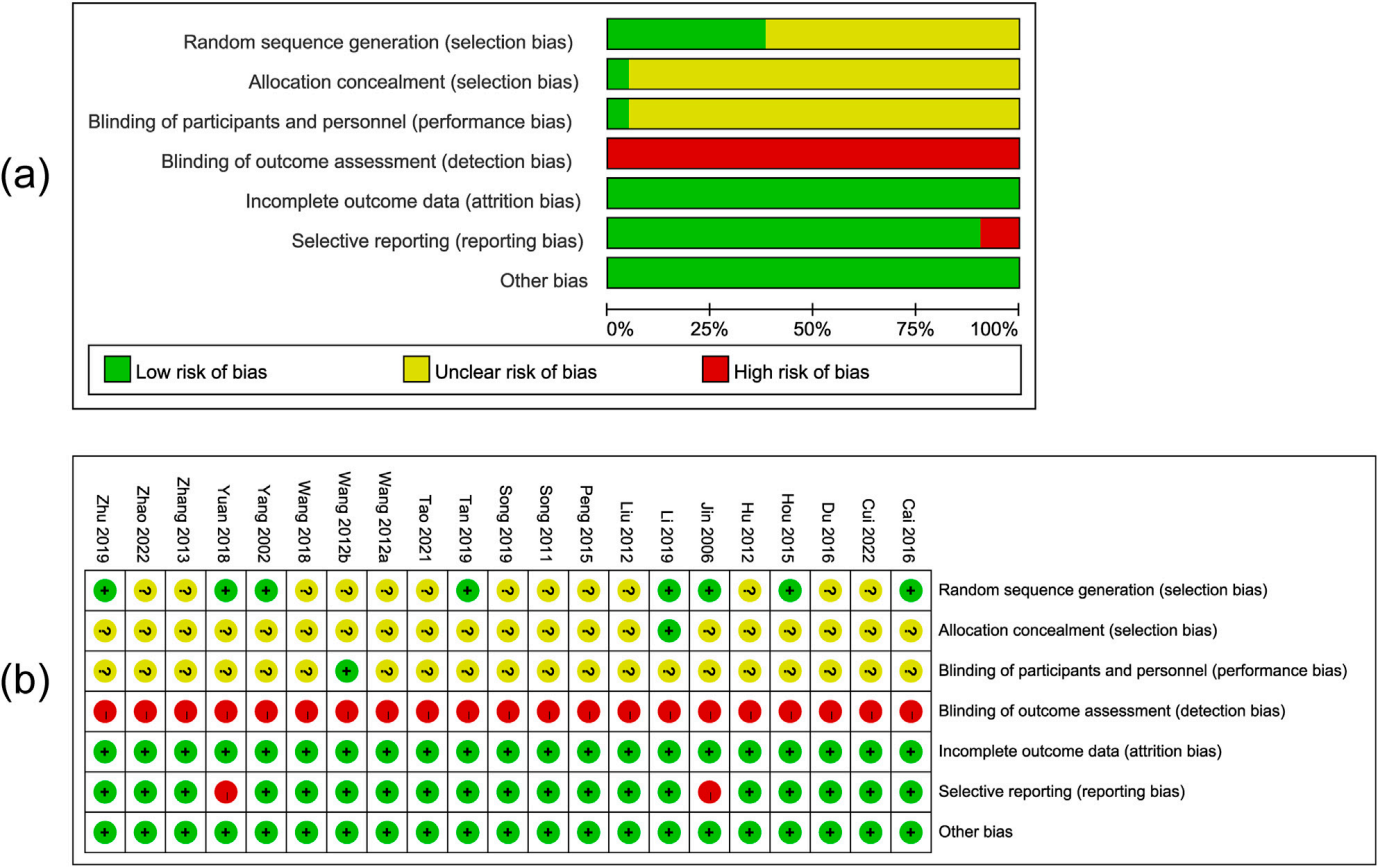
Risk of bias graph **(a)** and bias summary **(b)**. Note: Wang and Wang, 2012; Wang et al., 2012.

Two review authors evaluated the risk of bias of the included 21 RCTs and discrepancies were resolved via consensus. The results are shown in Figure 2. Among 21 RCTs, eight studies (Cai, 2016; Hou et al., 2015; Jin and Wu, 2006; Li et al., 2019; Tan, 2019; Yang and Zhou, 2002; Yuan, 2018; Zhu et al., 2019) presented a low risk of bias in the sequence generation process, one study (Li et al., 2019) presented a low risk of bias in allocation concealment, and one study (Wang et al., 2012) presented a low risk of bias in reporting blinding of participants. The studies did not describe blinding of outcome assessors and were thus judged as a high risk of bias. Twenty-one studies exhibited a low risk of attrition bias with complete outcome data. In terms of selective reporting bias, 19 trials (Cai, 2016; Cui, 2022; Du, 2016; Hou et al., 2015; Hu, 2012; Li et al., 2019; Liu, 2012; Peng, 2015; Song, 2011; Song, 2019; Tan, 2019; Tao, 2021; Wang and Wang, 2012; Wang et al., 2012; Wang and Sun, 2018; Yang and Zhou, 2002; Zhang, 2013; Zhao, 2022; Zhu et al., 2019) provided a low risk of bias and included all the outcomes specified in the methods section, while two trials (Jin and Wu, 2006; Yuan, 2018) showed a high risk of bias. We regarded all included studies as having a low risk of bias because we were unable to find any further sources of bias in any of them.

### 3.4 Description of TCM

In this study, sixteen TCM formulations were included, including Danxiong Tongluo decoction (*Salvia miltiorrhiza* Bunge (Danshen), *Pueraria montana* var. *lobata* (Willd.) Maesen & S.M.Almeida ex Sanjappa & Predeep (Gegen), *Crataegus monogyna* Jacq. (Shanzha), *Typha angustifolia* L. (Puhuang), *Oreocome striata* (DC.) Pimenov & Kljuykov (Chuanxiong), *Xanthium strumarium* L. (Gualoupi), *Allium chinense* G.Don (Xiebai), and *Pinellia ternata* (Thunb.) Makino (Banxia)), Tongqiao Huoxue decoction (*O. striata* (DC.) Pimenov & Kljuykov (Chuanxiong), *Carthamus tinctorius* L. (Honghua), *S. miltiorrhiza* Bunge (Danshen), *Paeonia lactiflora* Pall. (Chishao), *Juglans regia* L. (Taoren), *P. montana* var. *lobata* (Willd.) Maesen & S.M.Almeida ex Sanjappa & Predeep (Gegen), *Typha angustifolia* L. (Puhuang), *Astragalus mongholicus* Bunge (Huangqi), and *Codonopsis pilosula* (Franch.) Nannf. (Dangshen)), Wenxin granule (*C. pilosula* (Franch.) Nannf. (Dangshen), *Vitex negundo* L. (Huangjing), *Basella alba* L. (Sanqi), *Cannabis sativa* L. (Hupo), and *Nardostachys jatamansi* (D.Don) DC. (Gansong)), Danshen Dropping pill (*S. miltiorrhiza* Bunge (Danshen), *B. alba* L. (Sanqi), and *Camphora officinarum* Nees (Bingpian)), Tianma Gouteng Yin formula (*Gastrodia elata* Blume (Tianma), *Uncaria rhynchophylla* (Miq.) Miq. (Gouteng), *Prosthechea radiata* (Lindl.) W.E.Higgins (Shijueming), *Scutellaria baicalensis* Georgi (Huangqin), *Eucommia ulmoides* Oliv. (Duzhong), *Taxillus chinensis* (DC.) Danser (Sangjisheng), *Panax quinquefolius* L. (Xiyangshen), *Ziziphus jujuba* Mill. (Suanzaoren), *Panax ginseng* C.A.Mey. (Fushen), *S. miltiorrhiza* Bunge (Danshen), and *Achyranthes bidentata* Blume (Niuxi)), Shexiang Baoxin pill (*Liquidambar orientalis* Mill. (Shexiang), *P. ginseng* C.A.Mey. (Renshen), *P. ginseng* C.A.Mey. (Niuhuang), *Neolitsea cassia* (L.). Kosterm. (Rougui), *L. orientalis* Mill. (Suhexiang), *Tagetes erecta* L. (Chansu), and *C. officinarum* Nees (Bingpian)), Shengmai Yin (*P. ginseng* C.A.Mey. (Renshen), *Rehmannia glutinosa* (Gaertn.) DC. (Shudihuang), *Ophiopogon japonicus* (Thunb.) Ker Gawl. (Maidong), *Atractylodes macrocephala* Koidz. (Baizhu), *Schisandra chinensis* (Turcz.) Baill. (Wuweizi), *A. mongholicus* Bunge (Huangqi), *S. miltiorrhiza* Bunge (Danshen), and *Smilax glabra* Roxb. (Fuling)), Diju Pinggan capsule (Without reporting each botanical drug and dosage), and others. The botanical drug names have been checked with http://mpns.kew.org and http://www.worldfloraonline.org on 30 April 2024. The species involved have been taxonomically validated by searching their Latin names in the electronic version of Flora of China (http://www.efloras.org) to obtain descriptions of their morphological characteristics, type specimen information, and taxonomic status. Additionally, the original images of the type specimens of the species can be consulted through the International Plant Names Index (IPNI, https://www.ipni.org) or herbarium databases such as the Herbarium of the Institute of Botany, Chinese Academy of Sciences (PE, http://pe.ibcas.ac.cn). TCM formula Danxiong Tongluo decoction was the most commonly utilized (4/16, 25.00%), followed by Tongqiao Huoxue decoction (3/16, 18.75%).

Each Chinese botanical drug’s frequency in this review was described using a manual summary. There were 72 Chinese botanical drugs in all. The top five ranked Chinese botanical drugs were *S. miltiorrhiza* Bunge (Danshen) (16/72, 22.22%), *O. striata* (DC.) Pimenov & Kljuykov (Chuanxiong) (11/72, 15.28%), *P. montana* var. *lobata* (Willd.) Maesen & S.M.Almeida ex Sanjappa & Predeep (Gegen) (7/72, 9.72%), *A. mongholicus* Bunge (Huangqi) (7/72, 9.72%), and *Typha angustifolia* L. (Puhuang) (7/72, 9.72%). The provided formulations contained three to 14 Chinese botanical drugs. Among these formulas, Diju Pinggan capsule (batch number: 100,605) was provided by the pharmaceutical preparation room in Shanxi Academy of TCM without reporting each botanical drug and dosage.

Four dosage formulations of TCM reported, including decoction, pill, capsule, and granule, were all administered orally. The decoction was the most commonly used dosage formulation (17/21, 80.95%), followed by pill (2/21, 9.52%), granule (1/21, 4.76%), and capsule (1/21, 4.76%). The decoction was orally taken one dose every day. The TCM formulas and the specific botanical drugs are summarized concretely in Table 2.

**TABLE 2:** The botanical drugs of TCM used in the included studies.

References	Name of TCM	The botanical drugs of TCM
Cai (2016)	Danxiong Tongluo decoction	*Salvia miltiorrhiza* Bunge (Danshen) 30 g, *Pueraria montana* var. *lobata* (Willd.) Maesen & S.M.Almeida ex Sanjappa & Predeep (Gegen) 30 g, *Crataegus monogyna* Jacq. (Shanzha) 15 g, *Typha angustifolia* L. (Puhuang) 15 g, *Oreocome striata* (DC.) Pimenov & Kljuykov (Chuanxiong) 10 g, *Xanthium strumarium* L. (Gualoupi) 10 g, *Allium chinense* G.Don (Xiebai) 10 g, and *Pinellia ternata* (Thunb.) Makino (Banxia)10 g
Cui (2022)	Wenxin granule (Z10950026)	Chinese patent medicine: *Codonopsis pilosula* (Franch.) Nannf. (Dangshen), *Vitex negundo* L. (Huangjing), *Basella alba* L. (Sanqi), *Cannabis sativa* L. (Hupo), and *Nardostachys jatamansi* (D.Don) DC. (Gansong). 9 g three times a day
Du (2016)	Danshen Dropping pill (Z10950111)	Chinese patent medicine: *Salvia miltiorrhiza* Bunge (Danshen), *Basella alba* L. (Sanqi), and *Camphora officinarum* Nees (Bingpian). 270 mg three times a day
Hou et al. (2015)	TCM decoction	*Gastrodia elata* Blume (Tianma) 15 g, *Oreocome striata* (DC.) Pimenov & Kljuykov (Chuanxiong) 15 g, *Uncaria rhynchophylla* (Miq.) Miq. (Gouteng) 15 g, *Salvia miltiorrhiza* Bunge (Danshen) 15 g, *Achyranthes bidentata* Blume (Niuxi) 15 g, *Taxillus chinensis* (DC.) Danser (Sangjisheng) 9 g, *Plantago asiatica* L. (Cheqianzi) 9 g, *Epimedium sagittatum* (Siebold & Zucc.) Maxim. (Yinyanghuo) 9 g, *Ligustrum lucidum* W.T.Aiton (Nvzhenzi) 9 g, and *Glycyrrhiza glabra* L. (Gancao) 9 g
Hu (2012)	TCM decoction	*Neolitsea cassia* (L.) Kosterm. (Guizhi) 15 g, *Panax ginseng* C.A.Mey. (Renshen) 9 g, *Ophiopogon japonicus* (Thunb.) Ker Gawl. (Maidong) 12 g, *Lycium barbarum* L. (Gouqizi) 12 g, *Rehmannia glutinosa* (Gaertn.) DC. (Shudihuang) 12 g, *Angelica sinensis* (Oliv.) Diels (Danggui) 12 g, *Ziziphus jujuba* Mill. (Suanzaoren) 15 g, *Polygala tenuifolia* Willd. (Yuanzhi) 12 g, *Juglans regia* L. (Taoren) 12 g, and *Oreocome striata* (DC.) Pimenov & Kljuykov (Chuanxiong) 12 g
Jin and Wu (2006)	Tianma Gouteng Yin formula	*Gastrodia elata* Blume (Tianma) 10 g, *Uncaria rhynchophylla* (Miq.) Miq. (Gouteng) 20 g, *Prosthechea radiata* (Lindl.) W.E.Higgins (Shijueming) 15 g, *Scutellaria baicalensis* Georgi (Huangqin) 10 g, *Eucommia ulmoides* Oliv. (Duzhong) 15 g, *Taxillus chinensis* (DC.) Danser (Sangjisheng) 15 g, *Panax quinquefolius* L. (Xiyangshen) 6 g, *Ziziphus jujuba* Mill. (Suanzaoren) 20 g, *Panax ginseng* C.A.Mey. (Fushen) 10 g, *Salvia miltiorrhiza* Bunge (Danshen) 15 g, and *Achyranthes bidentata* Blume (Niuxi) 10 g
Li et al. (2019)	Danxiong Tongluo decoction	*Salvia miltiorrhiza* Bunge (Danshen) 30 g, *Pueraria montana* var. *lobata* (Willd.) Maesen & S.M.Almeida ex Sanjappa & Predeep (Gegen) 30 g, *Oreocome striata* (DC.) Pimenov & Kljuykov (Chuanxiong) 15 g, *Typha angustifolia* L. (Puhuang) 15 g, *Citrus × aurantium f. aurantium* (Zhishi) 15 g, *Crataegus monogyna* Jacq. (Shanzha) 15 g, *Basella alba* L. (Sanqi) 15 g, *Pinellia ternata* (Thunb.) Makino (Banxia) 10 g, *Allium chinense* G.Don (Xiebai) 10 g, *Xanthium strumarium* L. (Gualoupi) 10 g, and *Juglans regia* L. (Taoren) 10 g
Liu (2012)	TCM decoction	*Gastrodia elata* Blume (Tianma) 15 g, *Uncaria rhynchophylla* (Miq.) Miq. (Gouteng) 15 g, *Taxillus chinensis* (DC.) Danser (Sangjisheng) 12 g, *Ligustrum lucidum* W.T.Aiton (Nvzhenzi) 12 g, *Epimedium sagittatum* (Siebold & Zucc.) Maxim. (Yinyanghuo) 12 g, *Achyranthes bidentata* Blume (Niuxi) 15 g, *Salvia miltiorrhiza* Bunge (Danshen) 15 g, *Oreocome striata* (DC.) Pimenov & Kljuykov (Chuanxiong) 15 g, *Lathyrus sativus* L. (Guijia) 9 g, *Plantago asiatica* L. (Cheqianzi) 12 g, and *Glycyrrhiza glabra* L. (Gancao) 9 g
Peng (2015)	Shexiang Baoxin pill (Z31020068)	Chinese patent medicine: *Liquidambar orientalis* Mill. (Shexiang), *Panax ginseng* C.A.Mey. (Renshen), *Panax ginseng* C.A.Mey. (Niuhuang), *Neolitsea cassia* (L.) Kosterm. (Rougui), *Liquidambar orientalis* Mill. (Suhexiang), *Tagetes erecta* L. (Chansu), and *Camphora officinarum* Nees (Bingpian). 45 mg three times a day
Song (2011)	Yuyin Qianyang decoction	*Uncaria rhynchophylla* (Miq.) Miq. (Gouteng) 9 g, *Senna tora* (L.) Roxb. (Juemingzi) 30 g, *Prosthechea radiata* (Lindl.) W.E.Higgins (Shijueming) 30 g, *Cannabis sativa* L. (Muli) 30 g, *Zanthoxylum asiaticum* (L.) Appelhans, Groppo & J.Wen (Dilong) 9 g, *Xanthium strumarium* L. (Gualoupi) 15 g, *Pinellia ternata* (Thunb.) Makino (Banxia) 9 g, *Citrus reticulata* Blanco (Chenpi) 12 g, *Smilax glabra* Roxb. (Fuling) 15 g, *Salvia miltiorrhiza* Bunge (Danshen) 15 g, *Oreocome striata* (DC.) Pimenov & Kljuykov (Chuanxiong) 9 g, *Carthamus tinctorius* L. (Honghua) 6 g, *Rehmannia glutinosa* (Gaertn.) DC. (Dihuang) 12 g, and *Paeonia lactiflora* Pall. (Baishaoyao) 12 g
Song (2019)	TCM decoction	*Angelica sinensis* (Oliv.) Diels (Danggui) 30 g, *Scutellaria baicalensis* Georgi (Huangqin) 30 g, *Salvia miltiorrhiza* Bunge (Danshen) 15 g, *Gastrodia elata* Blume (Tianma) 15 g, *Epimedium sagittatum* (Siebold & Zucc.) Maxim. (Yinyanghuo) 12 g, *Paeonia lactiflora* Pall. (Baishaoyao) 12 g, *Lathyrus sativus* L. (Guijia) 9 g, *Achyranthes bidentata* Blume (Niuxi) 6 g, *Panax ginseng* C.A.Mey. (Renshen) 6 g, and *Asarum heterotropoides* F.Schmidt (Xixin) 5 g
Tan (2019)	Tongqiao Huoxue decoction	*Oreocome striata* (DC.) Pimenov & Kljuykov (Chuanxiong) 10 g, *Carthamus tinctorius* L. (Honghua) 9 g, *Salvia miltiorrhiza* Bunge (Danshen) 10 g, *Paeonia lactiflora* Pall. (Chishao) 10 g, *Juglans regia* L. (Taoren) 9 g, *Pueraria montana* var. *lobata* (Willd.) Maesen & S.M.Almeida ex Sanjappa & Predeep (Gegen) 10 g, *Typha angustifolia* L. (Puhuang) 10 g, *Astragalus mongholicus* Bunge (Huangqi) 20 g, and *Codonopsis pilosula* (Franch.) Nannf. (Dangshen) 15 g
Tao (2021)	TCM decoction	*Glycyrrhiza glabra* L. (Gancao) 6 g, *Pinellia ternata* (Thunb.) Makino (Banxia) 6 g, *Atractylodes macrocephala* Koidz. (Baizhu) 9 g, *Gastrodia elata* Blume (Tianma) 9 g, *Smilax glabra* Roxb. (Fuling) 12 g, *Arisaema erubescens* (Wall.) Schott (Dannanxing) 12 g, *Citrus reticulata* Blanco (Chenpi) 12 g, and Citrus × aurantium L. (Zhike) 12 g
Wang and Wang (2012)	Shengmai Yin	*Panax ginseng* C.A.Mey. (Renshen) 15–20 g, *Rehmannia glutinosa* (Gaertn.) DC. (Shudihuang) 20 g, *Ophiopogon japonicus* (Thunb.) Ker Gawl. (Maidong) 15 g, *Atractylodes macrocephala* Koidz. (Baizhu) 15 g, *Schisandra chinensis* (Turcz.) Baill. (Wuweizi) 10 g, *Astragalus mongholicus* Bunge (Huangqi) 30 g, *Salvia miltiorrhiza* Bunge (Danshen) 30 g, and *Smilax glabra* Roxb. (Fuling) 30 g
Wang et al. (2012)	Diju Pinggan capsule	Diju Pinggan capsule (batch number: 100,605) was provided by the pharmaceutical preparation room in Shanxi Academy of Traditional Chinese Medicine without reporting each botanical drug and dosage. 1.5 g three times a day
Wang and Sun (2018)	Tongqiao Huoxue decoction	*Paeonia lactiflora* Pall. (Chishao) 10 g, *Oreocome striata* (DC.) Pimenov & Kljuykov (Chuanxiong) 10 g, *Juglans regia* L. (Taoren) 9 g, *Ziziphus jujuba* Mill. (Dazao) 7, *Carthamus tinctorius* L. (Honghua) 9 g, *Andrographis paniculata* (Burm.f.) Wall. ex Nees (Cong) 3, *Zingiber officinale* Roscoe (Shengjiang) 9 g, *Liquidambar orientalis* Mill. (Shexiang) 0.15 g, *Salvia miltiorrhiza* Bunge (Danshen) 10 g, *Pueraria montana* var. *lobata* (Willd.) Maesen & S.M.Almeida ex Sanjappa & Predeep (Gegen) 10 g, *Typha angustifolia* L. (Puhuang) 10 g, *Astragalus mongholicus* Bunge (Huangqi) 20 g, and *Codonopsis pilosula* (Franch.) Nannf. (Dangshen) 15 g
Yang and Zhou (2002)	Danxiong Tongluo decoction	*Salvia miltiorrhiza* Bunge (Danshen) 30 g, *Oreocome striata* (DC.) Pimenov & Kljuykov (Chuanxiong) 10 g, *Pueraria montana* var. *lobata* (Willd.) Maesen & S.M.Almeida ex Sanjappa & Predeep (Gegen) 30 g, *Typha angustifolia* L. (Puhuang) 15 g, *Xanthium strumarium* L. (Gualoupi) 10 g, *Allium chinense* G.Don (Xiebai) 10 g, *Pinellia ternata* (Thunb.) Makino (Banxia) 10 g, and *Crataegus monogyna* Jacq. (Shanzha) 15 g
Yuan (2018)	Danxiong Tongluo decoction	*Xanthium strumarium* L. (Gualoupi) 9 g, *Pinellia ternata* (Thunb.) Makino (Banxia) 9 g, *Oreocome striata* (DC.) Pimenov & Kljuykov (Chuanxiong) 10 g, *Allium chinense* G.Don (Xiebai) 11 g, *Crataegus monogyna* Jacq. (Shanzha) 14 g, *Typha angustifolia* L. (Puhuang) 14 g, *Pueraria montana* var. *lobata* (Willd.) Maesen & S.M.Almeida ex Sanjappa & Predeep (Gegen) 28 g, and *Salvia miltiorrhiza* Bunge (Danshen) 28 g
Zhang (2013)	TCM decoction	*Panax ginseng* C.A.Mey. (Renshen) 6 g, *Astragalus mongholicus* Bunge (Huangqi) 30 g, *Angelica sinensis* (Oliv.) Diels (Danggui) 30 g, *Asarum heterotropoides* F.Schmidt (Xixin) 5 g, *Salvia miltiorrhiza* Bunge (Danshen) 15 g, *Gastrodia elata* Blume (Tianma) 15 g, *Paeonia lactiflora* Pall. (Baishaoyao) 12 g, *Achyranthes bidentata* Blume (Niuxi) 6 g, *Epimedium sagittatum* (Siebold & Zucc.) Maxim. (Yinyanghuo) 12 g, and *Lathyrus sativus* L. (Guijia) 9 g
Zhao (2022)	Tongqiao Huoxue decoction	*Codonopsis pilosula* (Franch.) Nannf. (Dangshen) 15 g, *Astragalus mongholicus* Bunge (Huangqi) 20 g, *Pueraria montana* var. *lobata* (Willd.) Maesen & S.M.Almeida ex Sanjappa & Predeep (Gegen) 10 g, *Typha angustifolia* L. (Puhuang) 10 g, *Salvia miltiorrhiza* Bunge (Danshen) 10 g, *Paeonia lactiflora* Pall. (Chishao) 10 g, *Oreocome striata* (DC.) Pimenov & Kljuykov (Chuanxiong) 10 g, *Juglans regia* L. (Taoren) 9 g, *Carthamus tinctorius* L. (Honghua) 9 g, *Zingiber officinale* Roscoe (Shengjiang) 9 g, *Liquidambar orientalis* Mill. (Shexiang) 0.15 g, *Andrographis paniculata* (Burm.f.) Wall. ex Nees (Cong) 3, and *Ziziphus jujuba* Mill. (Dazao) 7
Zhu et al. (2019)	Yiqi Wenyang Tongluo decoction	*Astragalus mongholicus* Bunge (Huangqi) 30 g, *Panax ginseng* C.A.Mey. (Hongshen) 15 g, *Cyperus rotundus* L. (Fuzi) 12 g, *Neolitsea cassia* (L.) Kosterm. (Guizhi) 10 g, *Carthamus tinctorius* L. (Honghua) 12 g, *Terminalia chebula* Retz. (Shuizhi) 5 g, *Salvia miltiorrhiza* Bunge (Danshen) 30 g, *Descurainia sophia* (L.) Webb ex Prantl (Tinglizi) 15 g, and *Eleutherococcus senticosus* (Rupr. & Maxim.) Maxim. (Wujiapi) 12 g

### 3.5 Primary outcomes

#### 3.5.1 BP

**FIGURE 3:**
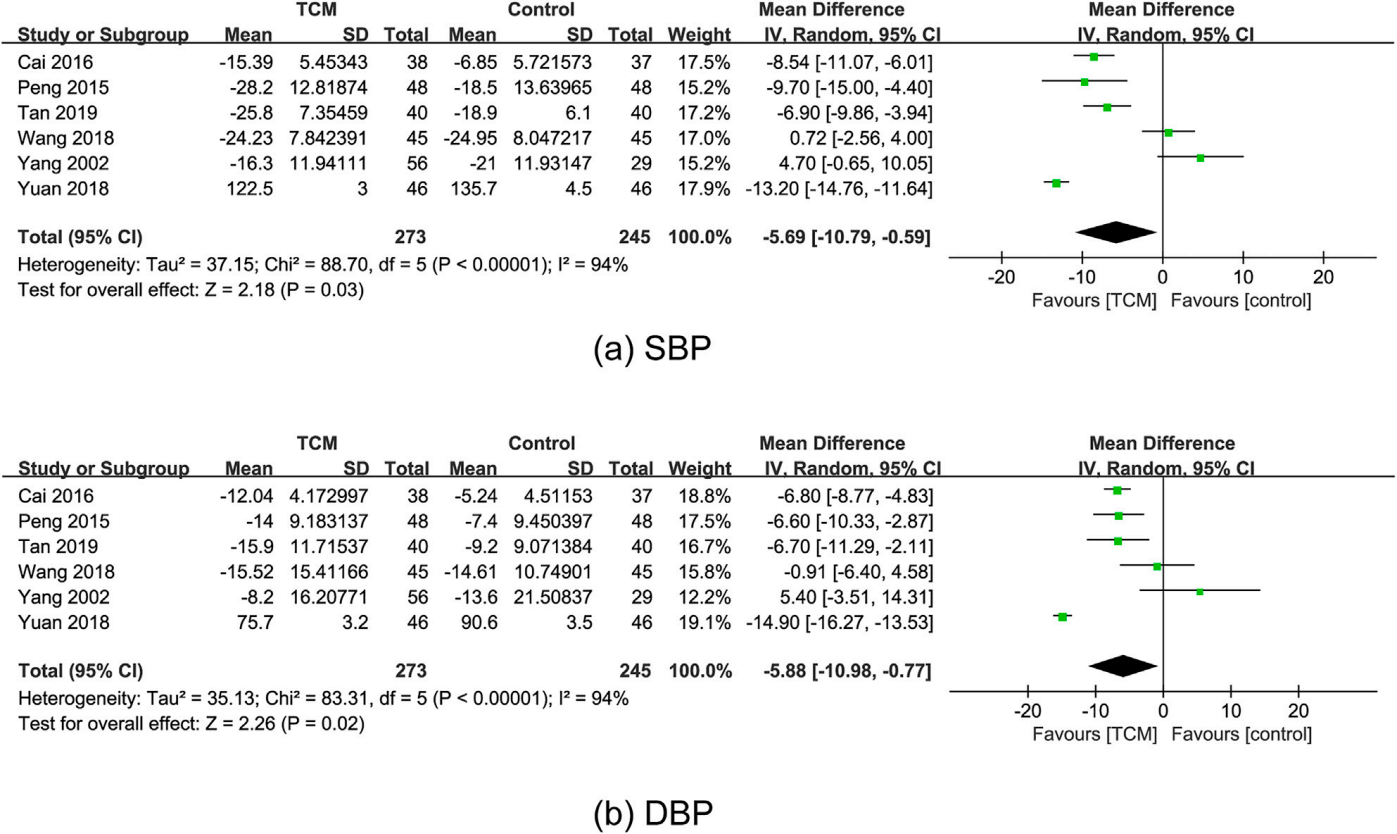
Forest plots for the meta-analysis of BP in the TCM group vs. the control group: **(a)** SBP and **(b)** DBP. Abbreviation: BP: blood pressure; DBP: diastolic blood pressure; SBP: systolic blood pressure; TCM: traditional Chinese medicine.

In total, six RCTs (Cai, 2016; Peng, 2015; Tan, 2019; Wang and Sun, 2018; Yang and Zhou, 2002; Yuan, 2018) reported the effect of TCM on BP. The results of the meta-analysis indicated that SBP was lower in the TCM group as compared to the control group (MD = −5.69; 95% CI: 10.79 to −0.59; P = 0.03), although the random-effects model exhibited statistical heterogeneity (χ^2^ = 88.70; I^2^ = 94%; P < 0.00001) (Figure 3a). Sensitivity analyses were performed to evaluate the robustness of the results. Thus, we repeated the meta-analysis after excluding, one by one, four studies (Cai, 2016; Peng, 2015; Tan, 2019; Yuan, 2018), P > 0.05 suggested nonsignificant difference and the unreliability of the result of SBP. In addition, another meta-analysis showed the efficacy of TCM on DBP (MD = −5.88; 95% CI: 10.98 to −0.77; P = 0.02), and also represented statistical heterogeneity (χ^2^ = 83.31; I^2^ = 94%; P < 0.00001) with the random-effects model (Figure 3b). The pooled effect estimates showed no significant difference for DBP after excluding Cai’s, Peng’s, and Tan’s studies (Cai, 2016; Peng, 2015; Tan, 2019) one by one, which suggested that the result was not robust.

#### 3.5.2 NYHA classification

**FIGURE 4:**
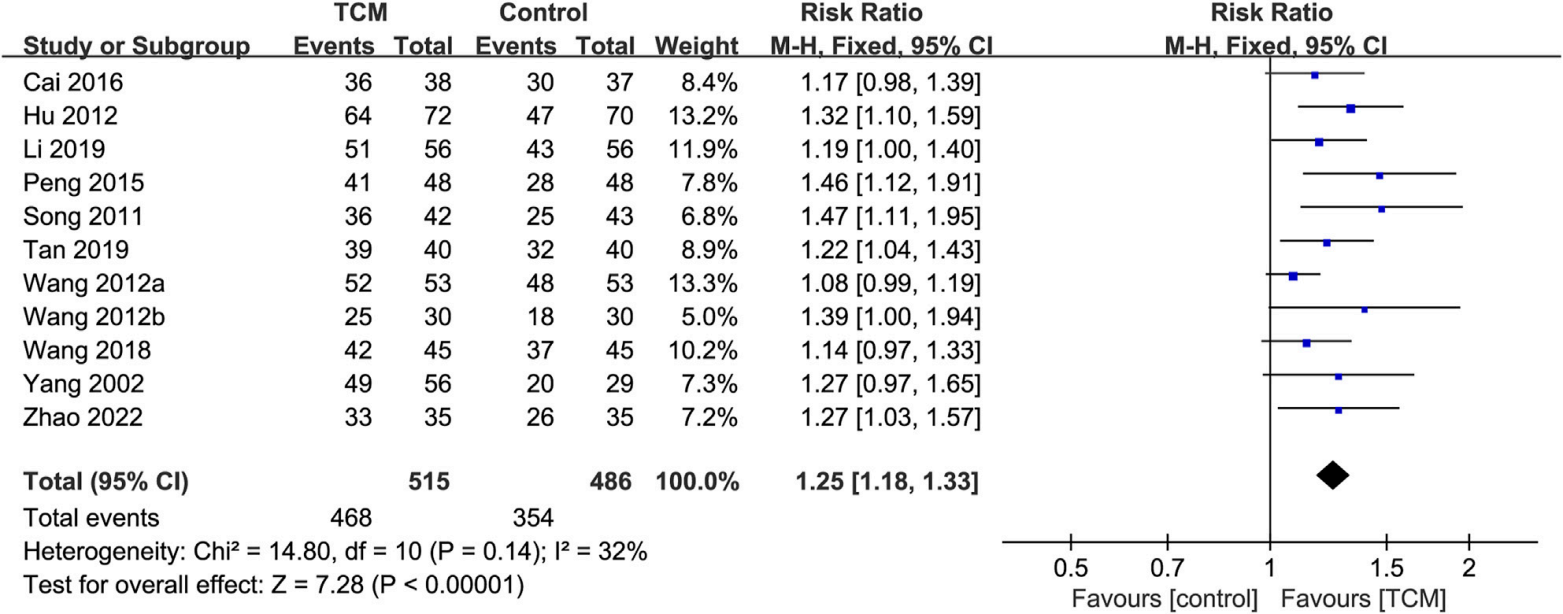
Forest plot for the meta-analysis of NYHA classification. Abbreviation: NYHA: New York Heart Association; TCM: traditional Chinese medicine. Note: Wang 2012a: Wang and Wang, 2012; Wang 2012b: Wang et al., 2012.

In total, eleven RCTs (Cai, 2016; Hu, 2012; Li et al., 2019; Peng, 2015; Song, 2011; Tan, 2019; Wang and Wang, 2012; Wang et al., 2012; Wang and Sun, 2018; Yang and Zhou, 2002; Zhao, 2022) reported the effect of TCM on NYHA classification. A meta-analysis revealed that the TCM group’s NYHA classification was substantially better than that of the control group (RR = 1.25; 95% CI: 1.18 to 1.33; P < 0.00001). There was moderate heterogeneity (χ^2^ = 14.80; I^2^ = 32%; P = 0.14) and the fixed-effects model was used (Figure 4). Sensitivity analyses indicated that the I^2^ dropped to 0% (P < 0.00001) by excluding Wang’s study (Wang and Wang, 2012), which might significantly impact the effect value and be the primary cause of heterogeneity.

#### 3.5.3 LVEF

**FIGURE 5:**
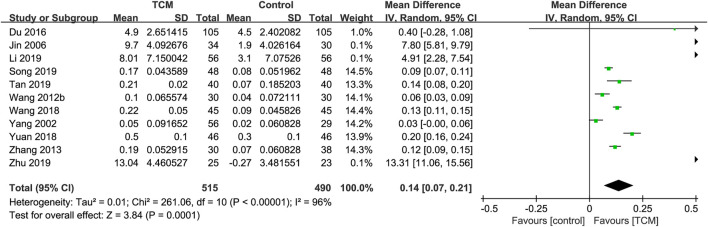
Forest plot for the meta-analysis of LVEF. Abbreviation: LVEF: left ventricular ejection fraction; TCM: traditional Chinese medicine. Note: Wang 2012b: Wang et al., 2012.

In total, thirteen RCTs (Cui, 2022; Du, 2016; Jin and Wu, 2006; Li et al., 2019; Song, 2019; Tan, 2019; Tao, 2021; Wang et al., 2012; Wang and Sun, 2018; Yang and Zhou, 2002; Yuan, 2018; Zhang, 2013; Zhu et al., 2019) reported the effect of TCM on LVEF. Two RCTs (Cui, 2022; Tao, 2021) were excluded because they did not measure or report baseline LVEF. The meta-analysis was performed with the 11 remaining studies containing 1, 005 patients (Du, 2016; Jin and Wu, 2006; Li et al., 2019; Song, 2019; Tan, 2019; Wang et al., 2012; Wang and Sun, 2018; Yang and Zhou, 2002; Yuan, 2018; Zhang, 2013; Zhu et al., 2019). The results of the meta-analysis indicated that LVEF of the TCM group was substantially greater than that of the control group (MD = 0.14; 95% CI: 0.07 to 0.21; P = 0.0001). However, the random-effects model exhibited statistical heterogeneity (χ^2^ = 261.06; I^2^ = 96%; P < 0.00001) (Figure 5). Sensitivity analyses demonstrated the robust results for LVEF.

### 3.6 Secondary outcomes

#### 3.6.1 CO

In total, two RCTs (Du, 2016; Tao, 2021) reported the effect of TCM on CO. One RCT (Tao, 2021) was excluded because it did not measure or report baseline CO. The meta-analysis was not performed due to insufficient available data. Only one study (Du, 2016) showed statistically significant difference in CO when comparing the TCM group to the control group. Given a lack of included trials, sensitivity analysis was not performed.

#### 3.6.2 E/A ratio

**FIGURE 6:**
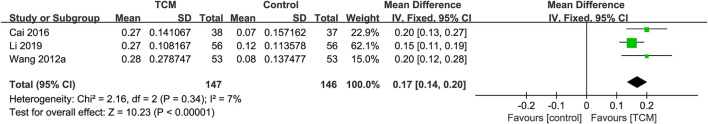
Forest plot for the meta-analysis of E/A ratio. Abbreviation: E/A: transmitral peak early diastolic velocity (E)/peak late diastolic velocity (A); TCM: traditional Chinese medicine. Note: Wang 2012a: Wang and Wang, 2012.

In total, three RCTs (Cai, 2016; Li et al., 2019; Wang and Wang, 2012) reported the effect of TCM on E/A ratio. According to a meta-analysis, the TCM group’s E/A ratio improved when compared to the control group (MD = 0.17; 95% CI: 0.14 to 0.20; P < 0.00001). There was low heterogeneity (χ^2^ = 2.16; I^2^ = 7%; P = 0.34) and the fixed-effects model was used (Figure 6). Sensitivity analyses demonstrated the robust results for E/A ratio.

#### 3.6.3 LVEDD

**FIGURE 7:**
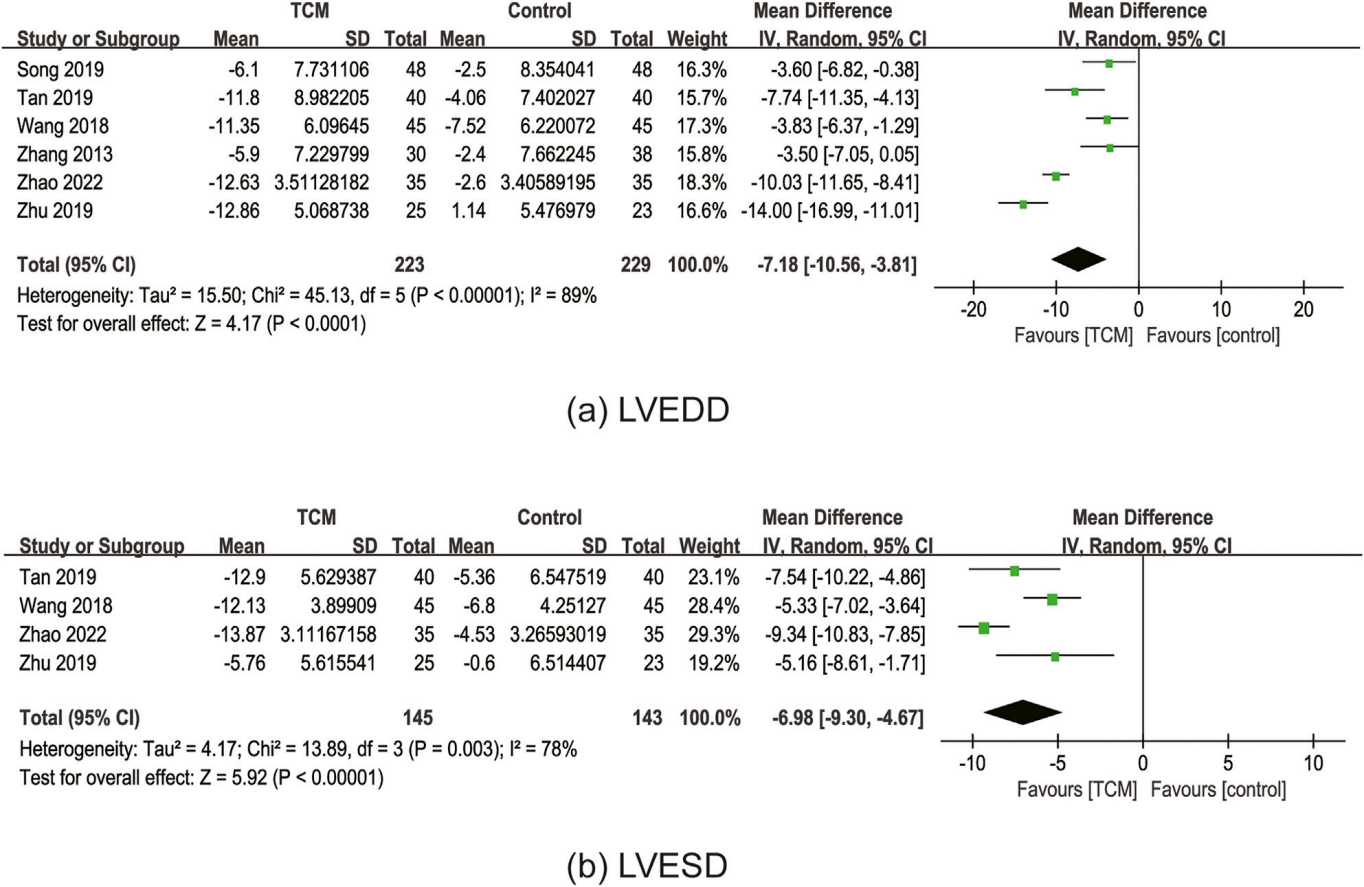
Forest plot for the meta-analysis of LVEDD **(a)** and LVESD **(b)**. Abbreviation: LVEDD: left ventricular end-diastolic diameter; LVESD: left ventricular end-systolic diameter; TCM: traditional Chinese medicine.

In total, nine RCTs (Hou et al., 2015; Liu, 2012; Song, 2019; Tan, 2019; Wang and Sun, 2018; Yuan, 2018; Zhang, 2013; Zhao, 2022; Zhu et al., 2019) reported the effect of TCM on LVEDD. Three RCTs (Hou et al., 2015; Liu, 2012; Yuan, 2018) were excluded because they did not measure or report baseline LVEDD. The meta-analysis was performed with the six remaining studies containing 452 patients (Song, 2019; Tan, 2019; Wang and Sun, 2018; Zhang, 2013; Zhao, 2022; Zhu et al., 2019). The results of the meta-analysis indicated that the TCM group’s LVEDD was considerably lower than that of the control group (MD = −7.18; 95% CI: 10.56 to −3.81; P < 0.0001). However, the random-effects model exhibited statistical heterogeneity (χ^2^ = 45.13; I^2^ = 89%; P < 0.00001) (Figure 7a). Sensitivity analyses demonstrated the robust results for LVEDD.

#### 3.6.4 LVESD

In total, four RCTs (Tan, 2019; Wang and Sun, 2018; Zhao, 2022; Zhu et al., 2019) reported the effect of TCM on LVESD. The results of the meta-analysis demonstrated that the TCM group’s LVESD was considerably lower than that of the control group (MD = −6.98; 95% CI: 9.30 to −4.67; P < 0.00001). However, the random-effects model indicated statistical heterogeneity (χ^2^ = 13.89; I^2^ = 78%; P = 0.003) (Figure 7b). Sensitivity analyses showed that the I^2^ dropped to 2% (P < 0.00001) by excluding Zhao’s study (Zhao, 2022), which might significantly impact the effect value and be the primary cause of heterogeneity.

#### 3.6.5 LVMI

**FIGURE 8:**
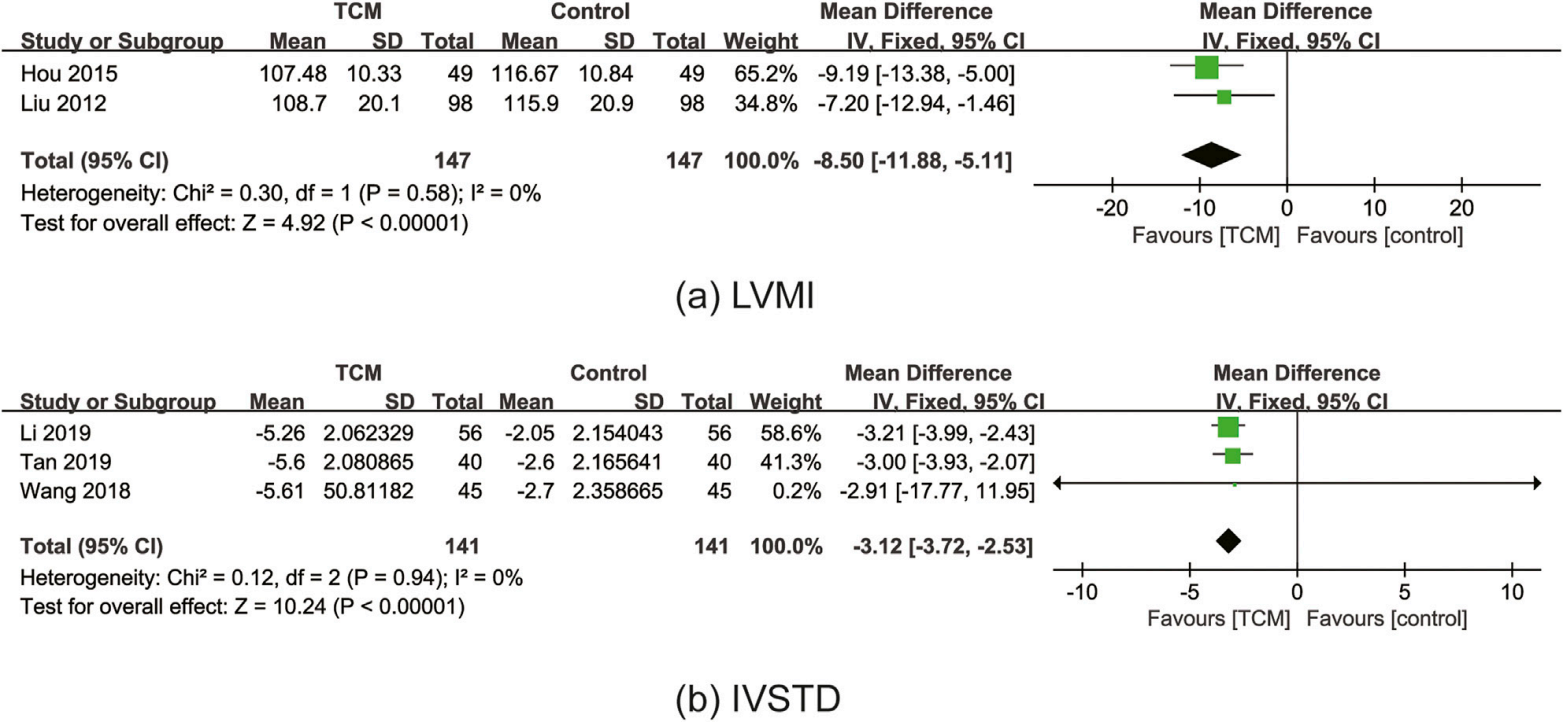
Forest plot for the meta-analysis of LVMI **(a)** and IVSTD **(b)**. Abbreviation: LVMI: left ventricular mass index; IVSTD: interventricular septum thickness in diastole; TCM: traditional Chinese medicine.

In total, two RCTs (Hou et al., 2015; Liu, 2012) reported the effect of TCM on LVMI. A meta-analysis revealed that the TCM group’s LVMI was considerably lower than that of the control group (MD = −8.50; 95% CI: 11.88 to −5.11; P < 0.00001). The fixed-effects model was applied, and there was low heterogeneity (χ^2^ = 0.30; I^2^ = 0%; P = 0.58) (Figure 8a). Given a lack of included trials, sensitivity analysis was not performed.

#### 3.6.6 IVSTD

In total, three RCTs (Li et al., 2019; Tan, 2019; Wang and Sun, 2018) reported the effect of TCM on IVSTD. A meta-analysis revealed that the TCM group’s IVSTD was considerably lower than that of the control group (MD = −3.12; 95% CI: 3.72 to −2.53; P < 0.00001). The fixed-effects model was applied, and there was low heterogeneity (χ^2^ = 0.12; I^2^ = 0%; P = 0.94) (Figure 8b). Sensitivity analyses demonstrated the robust results for IVSTD.

#### 3.6.7 BNP

**FIGURE 9:**
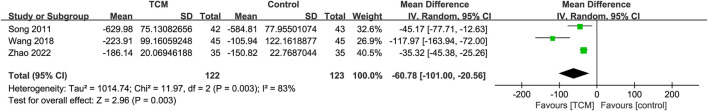
Forest plot for the meta-analysis of BNP. Abbreviation: BNP: B-type natriuretic peptide; TCM: traditional Chinese medicine.

In total, three RCTs (Song, 2011; Wang and Sun, 2018; Zhao, 2022) reported the effect of TCM on BNP. The results of the meta-analysis indicated that BNP was lower in the TCM group as compared to the control group (MD = −60.78; 95% CI: 101.00 to −20.56; P = 0.003), however, the random-effects model exhibited statistical heterogeneity (χ^2^ = 11.97; I^2^ = 83%; P = 0.003) (Figure 9). According to the results of the sensitivity analyses, after excluding Wang’s study (Wang and Sun, 2018), I^2^ dropped to 0% (P < 0.00001). In addition, after excluding Song’s study (Song, 2011), the statistically significant difference did not exist between the control group and the TCM group. These unrobust results showed that the meta-analysis of BNP was not reliable.

#### 3.6.8 Adverse events

**FIGURE 10:**
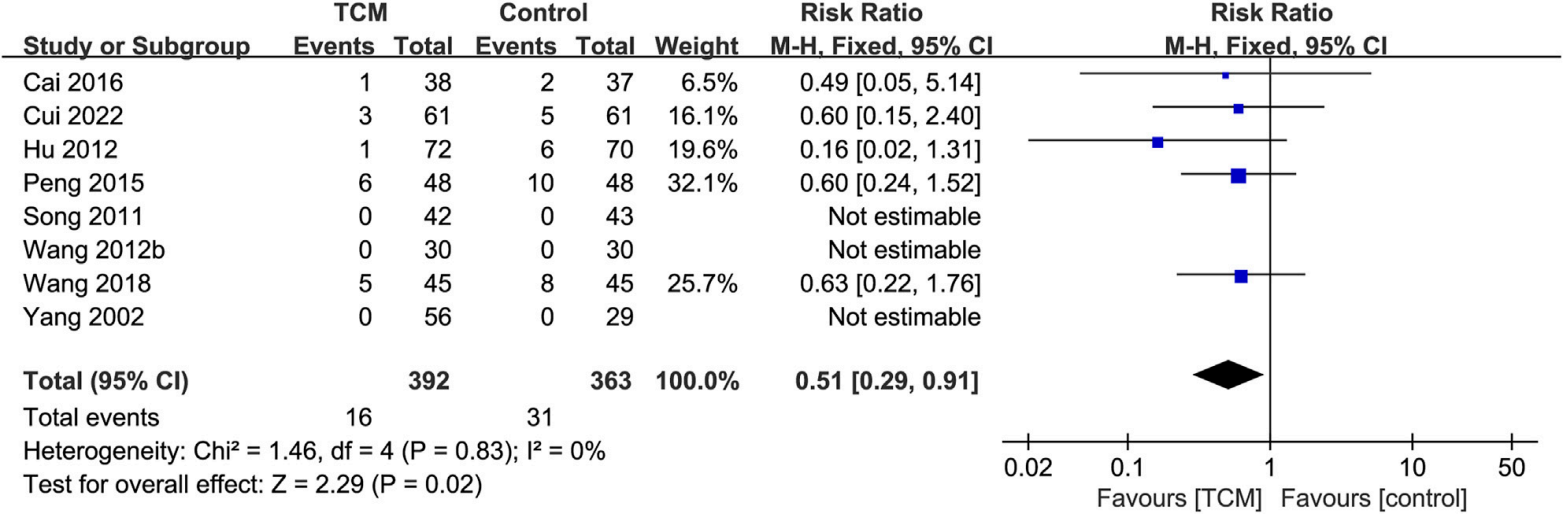
Forest plot for the meta-analysis of adverse events. Abbreviation: TCM: traditional Chinese medicine. Note: Wang 2012b: Wang et al., 2012.

In total, eight RCTs (Cai, 2016; Cui, 2022; Hu, 2012; Peng, 2015; Song, 2011; Wang et al., 2012; Wang and Sun, 2018; Yang and Zhou, 2002) reported adverse events, 16 in the TCM group, and 31 in the control group. According to a meta-analysis, individuals in the TCM group experienced fewer adverse events than those in the control group (RR = 0.51; 95% CI: 0.29 to 0.91; P = 0.02). The heterogeneity test showed low heterogeneity (χ^2^ = 1.46; I^2^ = 0%; P = 0.83), so the fixed-effects model was used (Figure 10). Sensitivity analyses revealed the unrobust results for adverse events. After excluding Hu’s study (Hu, 2012), the statistically significant difference did not exist between the control group and the TCM group. The most common adverse events included gastrointestinal symptoms, headache, arrhythmia, abnormal liver function, electrolyte imbalance, and respiratory failure. Of the eight studies, three studies (Song, 2011; Wang et al., 2012; Yang and Zhou, 2002) reported that there were no adverse events during the research period between the TCM and the control group.

### 3.7 Quality assessment of the evidence

The evidence profile was displayed in Table 3 and the degree of certainty of the evidence was evaluated using GRADEpro GDT. There was moderate-quality evidence on NYHA classification, E/A ratio, LVMI, IVSTD, and adverse events; low-quality evidence on LVEDD, LVESD, and BNP; and very low-quality evidence on BP (SBP and DBP) and LVEF. The most frequent sources of bias in randomized trials were the lack of blinding for study personnel and participants and the outcome assessment blinding. Thus, the ‘Risk of bias’ domain was judged to be at serious risk of bias. For the research inconsistency, there was high heterogeneity in six pieces of evidence, and the ‘Inconsistency’ domain was judged to be at serious inconsistency. Regarding the study’s imprecision, none of the indices went over the invalid line or received a downgrading. Regarding research indirectness, there was some inconsistency among the included studies in terms of interventions. However, there were no significant differences in their research purposes and no downgrades. The literature was carefully retrieved concerning publication bias, and no disclosed commercial conflicts of interest were present. In addition, publication bias was assessed for BP (SBP and DBP), NYHA classification, and LVEF. For BP (SBP and DBP) and LVEF, the assessment of publication bias was limited due to the insufficient number of studies for the outcomes. Thus, publication bias is strongly suspected.

**TABLE 3:** The GRADE evidence profile for TCM in the treatment of patients with HHD.

Certainty assessment							No of patients		Effect		Certainty	Importance
No of studies	Study design	Risk of bias	Inconsistency	Indirectness	Imprecision	Other considerations	TCM	Control	Relative (a95% CI)	Absolute (95% CI)		
SBP												
6	Randomized trials	Serious^a^	Serious^b^	Not serious	Not serious	Publication bias strongly suspected	273	245	-	MD 5.69 lower (10.79 lower to 0.59 lower)	⨁○○○ Very low	CRITICAL
DBP												
6	Randomized trials	Serious^a^	Serious^b^	Not serious	Not serious	Publication bias strongly suspected	273	245	-	MD 5.88 lower (10.98 lower to 0.77 lower)	⨁○○○ Very low	CRITICAL
NYHA classification												
11	Randomized trials	Serious^a^	Not serious	Not serious	Not serious	None	468/515 (90.9%)	354/486 (72.8%)	RR 1.25 (1.18–1.33)	182 more per 1,000 (from 131 more to 240 more)	⨁⨁⨁○ Moderate	CRITICAL
LVEF												
11	Randomized trials	Serious^a^	Serious^b^	Not serious	Not serious	Publication bias strongly suspected	515	490	-	MD 0.14 higher (0.07 higher to 0.21 higher)	⨁○○○ Very low	CRITICAL
E/A ratio												
3	Randomized trials	Serious^a^	Not serious	Not serious	Not serious	None^c^	147	146	-	MD 0.17 higher (0.14 higher to 0.2 higher)	⨁⨁⨁○ Moderate	IMPORTANT
LVEDD												
6	Randomized trials	Serious^a^	Serious^b^	Not serious	Not serious	None^c^	223	229	-	MD 7.18 lower (10.56 lower to 3.81 lower)	⨁⨁○○ Low	IMPORTANT
LVESD												
4	Randomized trials	Serious^a^	Serious^b^	Not serious	Not serious	None^c^	145	143	-	MD 6.98 lower (9.3 lower to 4.67 lower)	⨁⨁○○ Low	IMPORTANT
LVMI												
2	Randomized trials	Serious^a^	Not serious	Not serious	Not serious	None^c^	147	147	-	MD 8.5 lower (11.88 lower to 5.11 lower)	⨁⨁⨁○ Moderate	IMPORTANT
IVSTD												
3	Randomized trials	Serious^a^	Not serious	Not serious	Not serious	None^c^	141	141	-	MD 3.12 lower (3.72 lower to 2.53 lower)	⨁⨁⨁○ Moderate	IMPORTANT
BNP												
3	Randomized trials	Serious^a^	Serious^b^	Not serious	Not serious	None^c^	122	123	-	MD 60.78 lower (101 lower to 20.56 lower)	⨁⨁○○ Low	IMPORTANT
Adverse events												
8	Randomized trials	Serious^a^	Not serious	Not serious	Not serious	None^c^	16/392 (4.1%)	31/363 (8.5%)	RR 0.51 (0.29–0.91)	42 fewer per 1,000 (from 61 fewer to 8 fewer)	⨁⨁⨁○ Moderate	IMPORTANT

^*a*^ The quality of the majority of trials was not high. ^*b*^ Unexplained heterogeneity. ^*c*^ Funnel plots not completed due to*<*10 studies in the meta-analysis. BNP: B-type natriuretic peptide; CI: confidence interval; DBP: diastolic blood pressure; E/A: transmitral peak early diastolic velocity/peak late diastolic velocity; IVSTD: interventricular septum thickness in diastole; LVEDD: left ventricular end-diastolic diameter; LVEF: left ventricular ejection fraction; LVESD: left ventricular end-systolic diameter; LVMI: left ventricular mass index; MD: mean difference; NYHA: new york heart association; RR: risk ratio; SBP: systolic blood pressure; TCM, traditional Chinese medicine.

### 3.8 Publication bias

**FIGURE 11:**
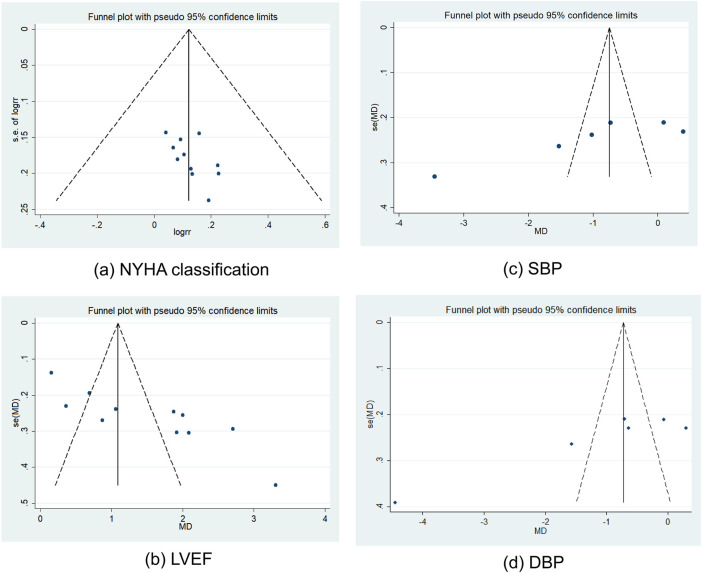
Funnel plots of NYHA classification **(a)**, LVEF **(b)**, SBP **(c)**, and DBP **(d)**. Abbreviation: DBP: diastolic blood pressure; LVEF: left ventricular ejection fraction; NYHA: New York Heart Association; SBP: systolic blood pressure.

The Egger’s test was used for the funnel plot to evaluate publication bias. There was no risk of publication bias in the NYHA classification, according to the symmetrical funnel plot (Figure 11a), whereas the asymmetric funnel plot implied a higher risk of publication bias in LVEF (Figure 11b), SBP (Figure 11c), and DBP (Figure 11d). The results of the meta-analysis were not significantly impacted by publication bias if the trim and fill strategy was applied (Table 4).

**TABLE 4:** The results of Egger’s test and trim and fill analysis.

Outcomes	Egger’s test		Trim and fill analysis
	T	P	
SBP	−3.39	0.027	MD = −1.312; 95% CI: 2.332 to −0.293; P = 0.012 < 0.05
DBP	−4.33	0.012	MD = −1.454; 95% CI: 2.553 to −0.355; P = 0.010 < 0.05
NYHA classification	2.11	0.064	—
LVEF	5.23	0.001	MD = 1.514; 95% CI: 0.946 to 2.081; P = 0.000 < 0.05

CI: confidence interval; DBP: diastolic blood pressure; LVEF: left ventricular ejection fraction; MD: mean difference; NYHA: new york heart association; SBP: systolic blood pressure.

## 4 Discussion

### 4.1 Summary of evidence

This meta-analysis included 21 studies that revealed the potential preventive effects of selected TCM as adjuvant therapy on HHD progression. In terms of BP, TCM as an adjunct in the treatment of hypertension could reduce BP to a certain extent. For the cardiac function, integrated TCM and WM significantly improved NYHA classification, LVEF, E/A ratio, and decreased LVEDD, LVESD, LVMI, IVSTD, and BNP, which indicated that TCM as adjuvant therapy played an important role in attenuating adverse LV remodeling and enhancing the heart’s diastolic and systolic functions to a certain extent. Furthermore, adverse events did not appear to be occurring more frequently linked to WM in the TCM group. In general, patients treated with TCM experienced fewer adverse events overall than those in comparator groups.

Vascular incidents were closely associated with elevated BP. A meta-analysis by Ettehad and colleagues (Ettehad et al., 2016) reported that a 10 mmHg decrease in SBP was associated with a 20% lower risk of major cardiovascular disease events, HF by 28%, and all-cause mortality by 13%. For patients having the greatest absolute risk of heart-related incidents, reducing BP would have the most overall benefits. This meta-analysis suggested that TCM as a potential adjuvant therapy effectively reduced BP. However, the results were not robust. It was necessary to conduct more research on the impact of TCM, especially in patients with LVH and hypertension.

The NYHA functional classification was used to assess symptom status, which was characterized as asymptomatic for NYHA Ⅰ and symptomatic for NYHA Ⅱ-Ⅳ (Egbe et al., 2020). This meta-analysis showed that TCM improved the NYHA classification, indicating that TCM could reduce symptoms and improve cardiac function in HHD patients.

Moreover, it was critical to observe the change in clinical, comprehensive imaging, and biomarker characteristics from simple hypertension to symptomatic HF with preserved ejection fraction (HFpEF) (Ekström et al., 2020). Echocardiography, one of the cardiac imaging modalities, was essential for quantifying changes in the heart structure and function without invasive methods as HF progressed. In terms of LV structure, the most intuitive markers of LV diastolic function were LV size as determined by LVEDD and LVESD. LVMI and IVSTD were used to assess LVH. LV systolic function was assessed using LVEF. CO was used to measure the strength and normality of cardiac ejection function. Due to sample size limitations, results on CO should be interpreted with caution. E/A ratio <1 indicated diastolic dysfunction. However, the E/A ratio was affected by age and decreased with older age, which influenced the accuracy of the result to a certain extent. According to the above, TCM as an adjunct played a significant role in the progression of cardiac hypertrophy and ventricular remodeling after HHD, which probably delayed the transition from HHD to HF.

Additionally, BNP could be used to screen patients with multiple HF risk factors and show a downward trend in HF and asymptomatic LV systolic dysfunction (Ledwidge et al., 2013; Slivnick and Lampert, 2019). This meta-analysis revealed that TCM combined with WM therapy effectively decreased BNP. Although the certainty of evidence about BNP was low and the result lacked robustness owing to the risk of bias, inconsistency, and insufficient data, this is an interesting proof-of-concept study that deserves further investigation (Slivnick and Lampert, 2019).

For the results with high heterogeneity, we explored several potential sources of heterogeneity, including the age of patients, the variations in TCM prescriptions, dosages, forms of dosage, modes of treatment, treatment duration, and different stages of HHD. These factors might also contribute to publication bias. However, subgroup age and TCM analysis were not possible owing to the small subgroup size. Some studies did not record the precise course of the disease, and none of the included RCTs mentioned the stage of HHD. In addition, despite doing additional sensitivity analyses to investigate the origins of heterogeneity, we were incapable of explaining the significant heterogeneity observed in the majority of our investigations. Thus, given the evidence overall ranged from moderate to very low certainty, care should be used when interpreting the findings.

### 4.2 The progression from hypertension to HHD and HF

Hypertension, characterized by raised systemic arterial pressure, is a chronic disease that has been considered an independent risk factor for cardiovascular disease and is associated with the development of HF (Liu et al., 2022; Ekström et al., 2020). Prolonged hypertension and the corresponding neurohormonal stimulation resulted in the malfunctioning of cardiomyocytes and the irregular build-up of cardiac extracellular matrix, which in turn caused cardiac fibrosis (Gülhan Mehmet et al., 2021; Mann and Felker, 2016; Salmas et al., 2017; Selamoglu Talas, 2014). Besides, the overactivated renin-angiotensin-aldosterone system (RAAS) and sympathetic nervous system also played an idiopathic role in cardiac fibrosis and LVH, which increased myocardial stiffness, caused aberrant myocardial systolic and diastolic function in the end by reducing ventricular compliance and restricting myocardial activity (Di Palo and Barone, 2020; Wright et al., 2008). HHD encompasses a spectrum of illnesses ranging from unmanaged hypertension to the ultimate development of HF (Slivnick and Lampert, 2019). Simple hypertension initiated the development of extracellular alterations and myocardial fibrosis, perhaps serving as a precursory mechanism in the development of HHD and HF from hypertension (Ekström et al., 2020). Diastolic dysfunction was thought to be the early developmental stage of HHD, and LVH was thought to be the trigger for the condition (Di Palo and Barone, 2020; Slivnick and Lampert, 2019). Persistent pressure overload in the heart due to persistent hypertension caused LVH and myocardial fibrosis, leading to progressive diastolic dysfunction, decompensation, increasing LV dilatation, and eccentric hypertrophy caused by sustained volume overload thereby causing systolic dysfunction to arise (Messerli et al., 2017). The heart is better protected and cardiac function is maintained in the early stages of cardiac hypertrophy (Hu et al., 2022; Bernardo et al., 2010). Prolonged hypertrophy, however, brought about inflammation, myocardial fibrosis, cardiomyocyte enlargement, and cardiac contractile dysfunction, all of which contributed to the development of chronic HF (Ritter and Neyses, 2003; Lieu and Koch, 2019; Hu et al., 2022). Once hypertensive LVH develops, the risk of developing heart failure, especially HFpHF, increases dramatically (Yao et al., 2017). Aggressive treatment might be able to reverse the development of LVH if it is identified early. However, the existence of LVH hastened the transition to HF and was irreversible once HF occurred (Slivnick and Lampert, 2019).

### 4.3 Pharmacological effects of TCM

**FIGURE 12:**
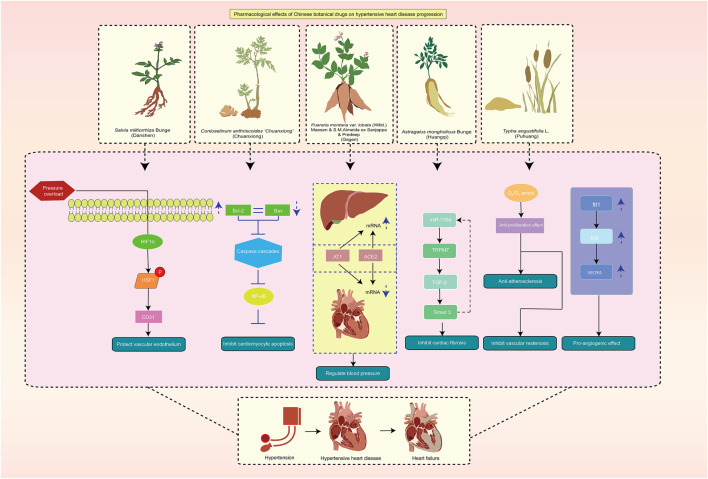
Pharmacological effects of TCM on HHD progression. Abbreviation: HHD: hypertensive heart disease; TCM: traditional Chinese medicine.

TCM, referred to as botanical medicine, phytomedicine, or phytotherapy, is the practice for medicinal purposes with the roots, seeds, bark, leaves, or flowers of plants, which is regarded as TCM in China. According to the World Health Organization (WHO) (WHO Traditional, 2021), botanical drugs, TCM preparations, and complete metabolites are considered to be part of TCM. The field of medicine has given TCM, as one of the complementary and alternative medicines, considerable attention, with a primary focus on active pharmaceutical metabolites. In this meta-analysis, the top 5 Chinese botanical drugs for replenishing qi and activating blood circulation were *S. miltiorrhiza* Bunge (Danshen), *O. striata* (DC.) Pimenov & Kljuykov (Chuanxiong), *P. montana* var. *lobata* (Willd.) Maesen & S.M.Almeida ex Sanjappa & Predeep (Gegen), *A. mongholicus* Bunge (Huangqi), and *Typha angustifolia* L. (Puhuang) (Figure 12). The five botanical drugs possess the effects of replenishing qi and activating blood circulation. They are used in the treatment of the development of HHD and HF from hypertension. Studies have shown that the method of replenishing qi and activating blood circulation can improve cardiac fibrosis after pressure overload-induced cardiac hypertrophy (Anwaier et al., 2022). The QiShenYiQi pill is a Chinese medicine approved by the China State Food and Drug Administration in 2003 for the treatment of cardiac dysfunction, and it includes *A. mongholicus* Bunge (Huangqi), *Panax notoginseng* (Burkill) F. H. Chen (Sanqi), *S. miltiorrhiza* Bunge (Danshen), and *Dalbergia odorifera* T. C. Chen (Jiangxiang). It inhibited myocardial fibrosis after pressure overload, which was mediated by ribosomal protein S19-mediated transforming growth factor β1 signaling and decreased four-and-a-half LIM domains protein 2 (Anwaier et al., 2022). The QiShenYiQi pill can also relieve fatigue-induced cardiac hypertrophy and enhance heart function, which is correlated with its potential to improve energy metabolism by regulating insulin-like growth factor-1 receptor signaling (Huang et al., 2019).

**FIGURE 13:**
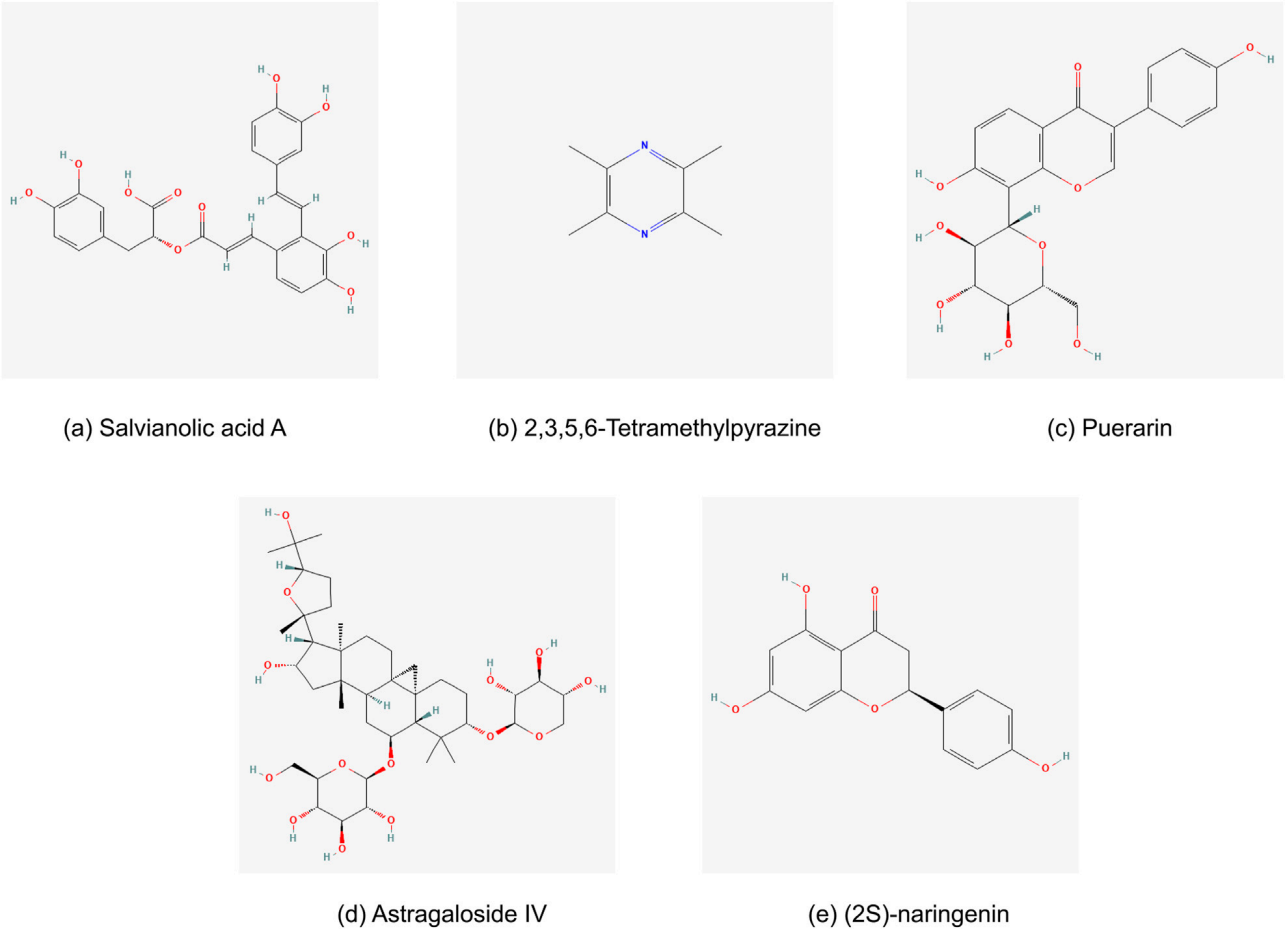
Chemical structures of main metabolites in the top 5 Chinese botanical drugs for the treatment of HHD: **(a)** Salvianolic acid A, **(b)** 2,3,5,6-Tetramethylpyrazine, **(c)** Puerarin, **(d)** Astragaloside IV, and **(e)** (2S)-naringenin.

*Salvia miltiorrhiza* Bunge (Danshen) was a traditional and precious Chinese botanical drug with high medicinal value, which was widely utilized to treat a variety of cardiovascular diseases. Clinically, *S. miltiorrhiza* Bunge (Danshen) could effectively enhance circulation, eliminate blood stasis, ameliorate inflammation, exert anti-oxidation, and inhibit vascular remodeling (Orgah et al., 2020). Maintaining endothelial function has been shown in earlier research to be a viable treatment approach for reducing pressure overload-induced heart damage (Wang et al., 2013; Su et al., 2015). Through endothelial protection, salvianolic acid, the main pharmacologic metabolites in *S. miltiorrhiza* Bunge (Danshen), reduced the effects of pressure overload-induced ventricular chamber expansion, cardiac dysfunction, and fibrosis. According to network pharmacology, salvianolic acid A (Figure 13a) was speculated the obstruct the important target proteins that mediate inflammatory responses such as apolipoprotein E, low-density lipoprotein cholesterol, and tumor necrosis factor, and protection for vascular endothelium in many ways (Sun et al., 2021). One experimental study in mice demonstrated that through an HIF1α/HSF1/CD31 pathway, salvianolic acid shielded cardiac endothelial cells from pressure overload, suggesting a possible use for salvianolic acid in HHD (Li N et al., 2022). In addition, A study revealed the role of salvianolic acid A in lowering cardiac fibrosis and hypertrophy in rats with spontaneous hypertension by inhibiting MMP-9 (Jiang et al., 2013). Neocryptotanshinone (NCTS) is a metabolite derived from *S. miltiorrhiza* Bunge (Danshen). It enhanced mitochondrial transcription factor A levels, promoted mitochondrial biogenesis, and increased myocardial adenosine triphosphate levels by activating retinoid X receptor α. The study has shown that NCTS improves myocardial energy metabolism, including fatty acid oxidation and mitochondrial biogenesis, by regulating the retinoid X receptor alpha α/peroxisome proliferator-activated receptor α pathway in mice with heart failure post-acute myocardial infarction (Ma et al., 2023).

*Oreocome striata* (DC.) Pimenov & Kljuykov (Chuanxiong) is a member of the Umbelliferae family and is grown mostly in Sichuan Province, China. It is a frequently prescribed TCM. In Shen Nong’s Materia Medica (Shen Nong Ben Cao Jing), *O. striata* (DC.) Pimenov & Kljuykov (Chuanxiong) could activate the blood, relieve pain, and remove blood stasis. The bioactive metabolites contained in *O. striata* (DC.) Pimenov & Kljuykov (Chuanxiong) primarily included alkaloids, phenols and organic acids, phthalides, and polysaccharides (Lin et al., 2022). Among these, Tetramethylpyrazine (Figure 13b) has been isolated as an alkaloid from the rhizome of *O. striata* (DC.) Pimenov & Kljuykov (Chuanxiong) and has multiple bioactivities (Yang et al., 2019). Tetramethylpyrazine has been shown in prior research to have a wide range of physiological effects, including protection against endothelial damage, antioxidative stress, anti-inflammatory, antiapoptotic, and antiplatelet aggregation, as well as improvements in microcirculation, vascular smooth muscle cell proliferation and migration, and vasodilation (Su et al., 2019; Lin et al., 2022). Besides, Liguzinediol, as a novel para-dihydroxy derivative of Tetramethylpyrazine extracted from the TCM Chuanxiong, demonstrated the effect on increasing heart function and preventing myocardial cell apoptosis, which was linked to controlling the expression of Bcl-s, Bax, caspase-s, and NF-κB expression in the rat model of HF (Li et al., 2014).

*Pueraria montana* var. *lobata* (Willd.) Maesen & S.M.Almeida ex Sanjappa & Predeep (Gegen) originated from Shen Nong’s Materia Medica (Shen Nong Ben Cao Jing) and is a notable TCM botanical drug. It is used to stimulate Spleen Yang to stop diarrhea and promote the production of bodily fluids. It has a sweet and acrid flavor (Wong et al., 2011). In clinical application, *P. montana* var. *lobata* (Willd.) Maesen & S.M.Almeida ex Sanjappa & Predeep (Gegen) is frequently used as a necessary botanical drug in TCM formulas to treat cardiovascular diseases, including hypertension, cardiac infarction, and angina pectoris. *Pueraria montana* var. *lobata* (Willd.) Maesen & S.M.Almeida ex Sanjappa & Predeep (Gegen) contains more than 70 metabolites, of which isoflavones and triterpenoids make up the majority. Puerarin (Figure 13c), the main bioactive metabolite and approximately 60% of all isoflavones, has a broad range of pharmacological characteristics, such as cardioprotection, vasodilation, anti-inflammatory effects, antioxidant activity, etc. (Zhang et al., 2020). Numerous animal models and cell cultures have shown puerarin’s pharmacological impacts on the cardiovascular system (Wong et al., 2011). A previous experiment revealed that puerarin inhibited β-adrenoceptors to provide its anti-hypertensive action (Lu et al., 1987). In another study, puerarin had a comparable impact to verapamil. Angiotensin Ⅱ type 1 receptor (AT1) and angiotensin-converting enzyme 2 (ACE2) mRNA expressions were considerably upregulated in hepatic tissues, while AT1 and ACE2 mRNA expressions in cardiac tissues were suppressed (Ye et al., 2008).

*Astragalus mongholicus* Bunge (Huangqi) is Chinese medicine with tonic, diuretic, blood-nourishing, and detoxifying properties recorded originally in Shen Nong’s Materia Medica (Shen Nong Ben Cao Jing) (Chinese Pharmacopoeia Commission, 2015). Previous study summarized that *A. mongholicus* Bunge (Huangqi) has obvious therapeutic effects on hypertension, cardiac hypertrophy, chronic HF, atherosclerosis, and other cardiovascular diseases (Li M et al., 2022). Furthermore, *A. mongholicus* Bunge (Huangqi) could strengthen myocardial contractility, protect myocardial cells, improve cardiac function, and increase myocardial energy metabolism (Lv et al., 2021; Chen et al., 2015). *Astragalus mongholicus* Bunge (Huangqi) contains various biological active metabolites, such as astragaloside, isoflavones, saponins, polysaccharides, and flavonoids. The primary mechanisms were anti-inflammatory, anti-oxidative damage, anti-apoptotic, immunomodulatory, and antithrombotic (Li et al., 2018). Astragaloside Ⅳ was one of the primary active metabolites of *A. mongholicus* Bunge (Huangqi) (Figure 13d). It has been found to target the miR-135a-TRPM7-TGF-β/Smads pathway, which may reduce cardiac fibrosis (Wei et al., 2020). Through the signaling pathways for ten-eleven translocation 2 and DNA methyltransferase 1, Astragaloside Ⅳ protects against vascular remodeling brought on by hypertension. This activity is crucial for controlling the function of vascular smooth muscle cells (Li M et al., 2022). In addition, Astragalus polysaccharides (ASP), which have therapeutic benefits on cardiovascular disorders such as cardiac hypertrophy and vascular endothelial dysfunction, were thought to be another significant metabolite of *A. mongholicus* Bunge (Huangqi). By blocking calcium-mediated calcineurin/NFATc3 and CaMKII signaling, ASP reduces cardiac hypertrophy in isoproterenol-induced hypertrophic myocardium (Chen et al., 2007). According to other animal and cell experiments, ASP has shown protective effectiveness in MVRI/ISO-treated cardiomyocytes by preventing apoptosis (Liu et al., 2018). ASP improved the pathological state of myocardial damage and chronic myocardial fibrosis by reducing the expression of inflammatory markers in the heart, including Interleukin-1β, interleukin-6, Tumor necrosis factor-α, monocyte chemoattractant protein-1, and interferon-γ (Liu et al., 2019). In TCM, Astragali Radix was often combined with other botanical drugs in various complex prescription formulas.

*Typha angustifolia* L. (Puhuang), the dried pollen of typha, was originally recorded in Shen Nong’s Materia Medica (Shen Nong Ben Cao Jing). The National Health Commission of the People’s Republic of China recognized it as a functional food in 2002, and the 2015 edition of the Pharmacopoeia of the People’s Republic of China included it (Gao et al., 2021). It was frequently used as TCM to treat angina pectoris, dysmenorrhea, hematuria, stranguria, stroke, metrorrhagia, and injuries from falls (Ding et al., 2018; Qin and Sun, 2005). *Typha angustifolia* L. (Puhuang) is mostly composed of flavonoids, sterols, amino acids, organic acids, long-chain hydrocarbons, and other chemicals (Ding et al., 2018). As is shown in pharmacological and clinical research, *Typha angustifolia* L. (Puhuang) is effective in improving microcirculation, raising cAMP levels, anti-inflammatory, antiplatelet aggregation, anti-atherosclerosis, anti-oxidant, preventing and treating hyperlipidemia, and coronary heart diseases (Qin and Sun, 2005; Hung and Wu, 2016; Chen et al., 2021; Ding et al., 2018). In cardiovascular effects, (2S)-naringenin (Figure 13e), as one of the active metabolites from *Typha angustifolia* L. (Puhuang), could suppress vascular smooth muscle cell proliferation induced platelet-derived growth factor receptor β through a G_0_/G_1_ arrest. This might be useful in managing vascular restenosis and atherosclerosis (Lee et al., 2012). And *Typha angustifolia* L. (Puhuang) also consisted mainly of the Korean herbal medicine Silsosangami. It reduced the expression of inducible nitric oxide synthase and cyclooxygenase-2, inhibited neutrophil activities, and produced prostaglandin E2 and nitric oxide. It also possessed anti-inflammatory properties (Park et al., 2004). Besides, another study demonstrated that *Typha angustifolia* L. (Puhuang) could upregulate the expression of kdr, flt1, and VEGFA to display the pro-angiogenic effect (Gao et al., 2021).

However, it should be acknowledged that the current attempt to clarify the pharmacological links between traditional therapeutic concepts and the findings has inherent limitations, which require systematic elaboration. This study primarily focuses on the concept of replenishing qi and activating blood circulation, and explores its potential association with specific pharmacological mechanisms, such as the regulation of energy metabolism pathways and the enhancement of immune function. To some extent, it reflects a reductionist tendency, which simplifies the complex, holistic traditional concept into measurable biological indicators, leading to an incomplete understanding of its connotations. Therefore, the findings of this study should be interpreted with caution. They only reflect a preliminary association between a certain pharmacological mechanism and one aspect of replenishing qi and activating blood circulation, rather than a comprehensive explanation. Future research needs to integrate multi-omics approaches, establish more systematic experimental models, and combine clinical syndrome differentiation data to further explore the complex links between traditional therapeutic concepts and modern pharmacology, thereby avoiding the narrow interpretation caused by over-reliance on reductionist methods. In the present study, replenishing qi and activating blood circulation emerge as a potential therapeutic approach for mitigating the progression of hypertensive heart disease. However, its efficacy, underlying mechanisms, and optimal clinical application scenarios warrant further in-depth investigation in future research to validate its therapeutic value and clarify its role within a broader context of treatment strategies.

### 4.4 Comparison to previous systematic review evidence

The differences from other systematic reviews (Mohammed et al., 2023; Ren et al., 2020; Xiong et al., 2019; Zhang et al., 2022) were given in the following three points. Firstly, in addition to BP, other necessary objective outcome measures, including NYHA classification, LVEF, CO, E/A ratio, LVEDD, LVESD, LVMI, IVSTD, BNP, and adverse events, were used to assess the effect of TCM on HHD. That’s the biggest difference compared to the previous research of TCM, which just focuses on hypertension. This meta-analysis focused on the impact of long-term hypertension on cardiac structure, function, and prognosis, with a particular emphasis on the progression from hypertension to HHD and HF, which had not been addressed in previous meta-analyses. Unfortunately, due to inadequate data in the included RCTs, long-term outcome endpoints such as cardiovascular death, HF incidence, hospitalization, and all-cause mortality were not investigated in this analysis. Secondly, the included studies did not place limitations on the TCM formula. Oral dose forms were the only available for TCM, including decoctions, pills, granules, and capsules. In contrast to conventional medicine, TCM has a long tradition of using food as medication. The third and fourth most often used Chinese botanical drugs in this meta-analysis, *P. montana* var. *lobata* (Willd.) Maesen & S.M.Almeida ex Sanjappa & Predeep (Gegen) and *A. mongholicus* Bunge (Huangqi), were found in the National Health Commission of the People’s Republic of China’s list of items that were ‘both food and medicine’ (also known as ‘medicine and food come from the same source’ or ‘medicine food homology,’ or MFH) (National Health Commission of the People’s Republic of China, 2023; National Health Commission of the People’s Republic of China, 2022). The other four Chinese botanical drugs were found in the National Health Commission of the People’s Republic of China’s list of Chinese medicines that could be used as health food. The efficacy of oral TCM for HHD was comprehensively evaluated, and its edible safety was well-guaranteed. Thirdly, other strengths, such as adherence to the guidelines of PRISMA and the previously registered protocol in PROSPERO, were also worth mentioning. The GRADE system was used to assess the quality of supporting evidence, and the Cochrane Risk of Bias Tool was utilized to evaluate the risk of bias in the included studies to facilitate the creation of recommendations.

### 4.5 Limitations

First, despite our thorough search, every included RCT was only done in China, which limited the generalizability. Future randomized, double-blind, placebo-controlled RCTs with a longer-term duration are required. Second, this meta-analysis was subjected to methodological weaknesses of the original studies. Only one study reported allocation concealment that could result in selection bias, and one study described blinding of participants and personnel that could cause performance bias. Blinding of outcome assessment was not mentioned in any of the studies, which could result in detection bias. The majority of the included studies’ low quality had an impact on the accuracy of the results. Subsequent research endeavors ought to incorporate methods that mitigate the possibility of bias in reporting, like blinding result assessors, randomization, and allocation concealment. However, because TCM consists of many metabolites, conducting adequate blinding for TCM investigations may prove challenging. Moreover, many ancient forms, such as decoctions, pills, and powders, had special tastes and scents, which made it difficult to confirm that placebos were identical. More information is needed on the reported side effects, interactions, and general safety aspects of this preparation. Third, the onset and progression of HHD are complicated processes. To date, there are no guidelines or expert consensus providing recommendations on the stage of HHD. It is still difficult to recognize patients with hypertension at risk of developing HF in the long run promptly, even with the availability of targeted antihypertensive medications. Finally, because the included studies only provided a limited number of outcome indicators, this article was unable to assess the impact of TCM on HHD in its entirety. Thus, it is necessary to extend the follow-up time and conduct an RCT to identify the efficacy of TCM on HHD and observe the occurrence of long-term cardiovascular adverse events.

### 4.6 Future perspectives

The analysis we had done to reflect the changes in heart structure and function following HHD, but it was still not enough to show how this intricate shift in the development of HHD and HF occurs after hypertension. Further research should observe blood indicators for endothelial dysfunction, inflammation, and cardiac fibrosis, which could be analyzed to describe, track, and identify phenotypes that are at risk of developing HHD and HFpEF (Ekström et al., 2020). Additionally, TCM has been demonstrated to be an alternate and complementary strategy for both primary and secondary cardiovascular disease prevention (Hao et al., 2017). Typically, two or more botanical drugs are combined in a TCM recipe to create a synergistic effect. Botanical drug or botanical drug-pair interactions should be closely monitored from a clinical standpoint, particularly when several botanical drugs are utilized at once (Zuo et al., 2020). Our goal is to find new hypertension treatment strategies to prevent HF. More RCTs are required to evaluate how therapy of replenishing qi and activating blood circulation affects patients with HHD’s long-term challenging endpoints. The results are expected to guide healthcare providers in hospitals to offer personalized treatment (Ekström et al., 2020).

## 5 Conclusion

In general, the results of this meta-analysis suggested that the use of TCM and WM together may be more effective than using WM alone in the treatment of HHD. This combination might also reduce unfavorable LV remodeling and enhance cardiac systolic and diastolic function, which might slow the disease’s progression. In addition to being a new approach to treating hypertension to avoid HF, therapy of replenishing qi and activating blood circulation offers a reference as an auxiliary treatment for secondary prevention following HHD. However, it was important to interpret these results cautiously, considering the limitations of the original trials. To support this clinical evidence, more rigorous trials for herbal therapy are advised.

## 6 Chemical metabolites studied in this article

Salvianolic acid A (PubChem CID: 5281793); 2,3,5,6-Tetramethylpyrazine (PubChem CID: 14296); Puerarin (PubChem CID: 5281807); Astragaloside IV (PubChem CID: 13943297); (2S)-naringenin (PubChem CID: 439246).

## Data Availability

The original contributions presented in the study are included in the article/Supplementary Material, further inquiries can be directed to the corresponding authors.

## Glossary

ACE2: angiotensin-converting enzyme 2
ASP: Astragalus polysaccharides
AT1: angiotensin Ⅱ type 1 receptor
BNP: B-type natriuretic peptide
BP: blood pressure
C: the control group
CI: confidence interval
CNKI: China National Knowledge Infrastructure
CO: cardiac output
DBP: diastolic blood pressure
E/A: transmitral peak early diastolic velocity (E)/peak late diastolic velocity (A)
GDT: Guideline Development Tool
GRADE: Grading of Recommendations Assessment Development and Evaluation
HF: heart failure
HFpEF: heart failure with preserved ejection fraction
HHD: hypertensive heart disease
HM: herbal medicine
IVSTD: interventricular septum thickness in diastole
LV: left ventricular
LVEDD: left ventricular end-diastolic diameter
LVEF: left ventricular ejection fraction
LVESD: left ventricular end-systolic diameter
LVH: left ventricular hypertrophy
LVMI: left ventricular mass index
MD: mean difference
NR: not reported
NYHA: New York Heart Association
PRISMA: Preferred Reporting Items for Systematic Reviews and Meta-Analyses
RAAS: renin-angiotensin-aldosterone system
RCTs: randomized controlled trials
RR: risk ratio
SBP: systolic blood pressure
SinoMed: Chinese Biomedical Database
SMD: standardized mean difference
T: the TCM group
TCM: traditional Chinese medicine
VIP: Chinese Scientific Journal Database (Chinese VIP Information)
WHO: World Health Organization
WM: Western medicine

## References

[B1] Anwaier G. Xie T. T. Pan C. S. Li A. Q. Yan L. Wang D. (2022). QiShenYiQi pill ameliorates cardiac fibrosis after pressure overload-induced cardiac hypertrophy by regulating FHL2 and the macrophage RP S19/TGF-β1 signaling pathway. Front. Pharmacol. 13, 918335. 10.3389/fphar.2022.918335 35910357 PMC9326396

[B2] Bayés-Genís A. Díez J. (2022). Transition to heart failure in hypertension: going to the heart of the matter. Eur. Heart J. 43 (35), 3332–3334. 10.1093/eurheartj/ehab651 34516629

[B3] Bernardo B. C. Weeks K. L. Pretorius L. McMullen J. R. (2010). Molecular distinction between physiological and pathological cardiac hypertrophy: experimental findings and therapeutic strategies. Pharmacol. Ther. 128 (1), 191–227. 10.1016/j.pharmthera.2010.04.005 20438756

[B4] Cai F. (2016). Clinical observation of 38 cases of hypertensive heart disease complicated with left ventricular diastolic dysfunction treated with traditional Chinese medicine decoction. Cardiovasc. Dis. Prev. Knowl. Acad. Ed. 22, 8–10.

[B5] Chen W. Li Y. M. Yu M. H. (2007). Effects of Astragalus polysaccharides on chymase, angiotensin-converting enzyme and angiotensin II in diabetic cardiomyopathy in hamsters. J. Int. Med. Res. 35 (6), 873–877. 10.1177/147323000703500615 18035000

[B6] Chen W. Lai Y. Wang L. Xia Y. Chen W. Zhao X. (2015). Astragalus polysaccharides repress myocardial lipotoxicity in a PPARalpha-dependent manner *in vitro* and *in vivo* in mice. J. Diabetes Complicat. 29 (2), 164–175. 10.1016/j.jdiacomp.2014.11.007 25499591

[B7] Chen T. Chen C. Huang Y. Baskaran R. Tsai J. J. P. Hu R. (2021). Ethanolic extract of Puhuang (Pollen Typhae) modulates lipopolysaccharide-induced inflammatory response through inducible nitric oxide synthase/cyclooxygenase-2 signaling in RAW 264.7 macrophage. J. Tradit. Chin. Med. 41 (6), 836–844. 10.19852/j.cnki.jtcm.2021.06.002 34939379

[B8] Chinese Pharmacopoeia Commission (2015). Pharmacopoeia of the People’s Republic of China. Beijing: China Medical Science and Technology Press.

[B9] Cui J. (2022). Clinical observation of metoprolol combined with Wenxin granule in the treatment of hypertensive heart disease with premature ventricular beat. Database Chin. Sci-tech Period. (Med.). 4, 0039–0042.

[B10] Dai H. Bragazzi N. L. Younis A. Zhong W. Liu X. Wu J. (2021). Worldwide trends in prevalence, mortality, and disability-adjusted Life years for hypertensive heart disease from 1990 to 2017. Hypertension 77 (4), 1223–1233. 10.1161/HYPERTENSIONAHA.120.16483 33583201

[B11] Devereux R. B. Koren M. J. de Simone G. Okin P. M. Kligfield P. (1993). Methods for detection of left ventricular hypertrophy: application to hypertensive heart disease. Eur. Heart J. 14 (Suppl. D), 8–15. 10.1093/eurheartj/14.suppl_d.8 8370376

[B12] Di Palo K. E. Barone N. J. (2020). Hypertension and heart failure: prevention, targets, and treatment. Heart fail. Clin. 16 (1), 99–106. 10.1016/j.hfc.2019.09.001 31735319

[B13] Ding M. Jiang Y. Yu X. Zhang D. Li J. Wang H. (2018). Screening of combinatorial quality markers for natural products by metabolomics coupled with chemometrics. A case study on pollen typhae. Front. Pharmacol. 9, 691. 10.3389/fphar.2018.00691 30002628 PMC6033115

[B14] Drazner M. H. (2011). The progression of hypertensive heart disease. Circulation 123 (3), 327–334. 10.1161/CIRCULATIONAHA.108.845792 21263005

[B15] Du H. (2016). Analysis of clinical effect of integrated traditional Chinese and Western medicine on hypertensive heart disease. Cardiovasc. Dis. J. Integr. Tradit. Chin. West. Med. 4 (34), 181.

[B16] Egbe A. C. Anderson J. H. Ammash N. M. Taggart N. W. (2020). Left ventricular remodeling after transcatheter versus surgical therapy in adults with coarctation of aorta. JACC Cardiovasc. Imaging. 13 (9), 1863–1872. 10.1016/j.jcmg.2020.01.016 32199847 PMC7486991

[B17] Ekhteiari Salmas R. Durdagi S. Gulhan M. F. Duruyurek M. Abdullah H. I. Selamoglu Z. (2018). The effects of pollen, propolis, and caffeic acid phenethyl ester on tyrosine hydroxylase activity and total RNA levels in hypertensive rats caused by nitric oxide synthase inhibition: experimental, docking and molecular dynamic studies. J. Biomol. Struct. Dyn. 36 (3), 609–620. 10.1080/07391102.2017.1288660 28132600

[B18] Ekström M. Hellman A. Hasselström J. Hage C. Kahan T. Ugander M. (2020). The transition from hypertension to hypertensive heart disease and heart failure: the PREFERS Hypertension study. Esc. Heart Fail 7 (2), 737–746. 10.1002/ehf2.12612 32073753 PMC7160482

[B19] Escaned J. Lerman L. O. (2020). Coronary microcirculation and hypertensive heart failure. Eur. Heart J. 41 (25), 2376–2378. 10.1093/eurheartj/ehaa437 32608497

[B20] Ettehad D. Emdin C. A. Kiran A. Anderson S. G. Callender T. Emberson J. (2016). Blood pressure lowering for prevention of cardiovascular disease and death: a systematic review and meta-analysis. Lancet 387 (10022), 957–967. 10.1016/S0140-6736(15)01225-8 26724178

[B21] Gao M. Ge Z. Deng R. Bao B. Yao W. Cao Y. (2021). Evaluation of VEGF mediated pro-angiogenic and hemostatic effects and chemical marker investigation for Typhae Pollen and its processed product. J. Ethnopharmacol. 268, 113591. 10.1016/j.jep.2020.113591 33212176

[B22] GBD 2017 Risk Factor Collaborators (2018). Global, regional, and national comparative risk assessment of 84 behavioural, environmental and occupational, and metabolic risks or clusters of risks for 195 countries and territories, 1990-2017: a systematic analysis for the Global Burden of Disease Study 2017. Lancet 392 (10159), 1923–1994. 10.1016/S0140-6736(18)32225-6 30496105 PMC6227755

[B23] Gogebakan A. Talas Z. S. Ozdemir I. Sahna E. (2012). Role of propolis on tyrosine hydroxylase activity and blood pressure in nitric oxide synthase-inhibited hypertensive rats. Clin. Exp. Hypertens. 34 (6), 424–428. 10.3109/10641963.2012.665542 22471835

[B24] González A. Ravassa S. López B. Moreno M. U. Beaumont J. San José G. (2018). Myocardial remodeling in hypertension. Hypertension 72 (3), 549–558. 10.1161/HYPERTENSIONAHA.118.11125 30354762

[B25] Gülhan Mehmet F. Özdemir B. Selamoglu Z. Şahna E. (2021). The effects of apitherapeutic agents on oxidative stress in serum metabolic parameters of hypertensive rats created by nitric oxide synthase inhibited. Sains Malays. 50 (6), 1745–1754. 10.17576/jsm-2021-5006-20

[B26] Hao P. Jiang F. Cheng J. Ma L. Zhang Y. Zhao Y. (2017). Traditional Chinese medicine for cardiovascular disease: evidence and potential mechanisms. J. Am. Coll. Cardiol. 69 (24), 2952–2966. 10.1016/j.jacc.2017.04.041 28619197

[B27] Higgins J. P. Thompson S. G. Deeks J. J. Altman D. G. (2003). Measuring inconsistency in meta-analyses. BMJ 327 (7414), 557–560. 10.1136/bmj.327.7414.557 12958120 PMC192859

[B28] Higgins J. P. Altman D. G. Gøtzsche P. C. Jüni P. Moher D. Oxman A. D. (2011). The Cochrane Collaboration's tool for assessing risk of bias in randomised trials. BMJ 343, d5928. 10.1136/bmj.d5928 22008217 PMC3196245

[B29] Hou B. Liu X. Song Y. Zhang T. (2015). Clinical study of 98 patients with hypertensive heart disease treated by combination of Chinese and Western medicine. Glob. Tradit. Chin. Med. 8 (S1), 242.

[B30] Hu X. (2012). Clinical observation of patients with hypertensive heart disease treated by combination of traditional Chinese and Western medicine. World Health Dig. Med. Period. 36, 184–185. 10.3969/j.issn.1672-5085.2012.36.172

[B31] Hu L. Wei J. Zhang Y. Wang Z. Tang J. Tang J. (2022). ANGPTL8 is a negative regulator in pathological cardiac hypertrophy. Cell Death Dis. 13 (7), 621. 10.1038/s41419-022-05029-8 35851270 PMC9293964

[B32] Huang R. Cui Y. C. Wei X. H. Pan C. S. Li Q. He S. Y. (2019). A novel traditional Chinese medicine ameliorates fatigue-induced cardiac hypertrophy and dysfunction via regulation of energy metabolism. Am. J. Physiol. Heart Circ. Physiol. 316 (6), H1378–H1388. 10.1152/ajpheart.00731.2018 30951366

[B33] Hung H. Y. Wu T. S. (2016). Recent progress on the traditional Chinese medicines that regulate the blood. J. Food Drug Anal. 24 (2), 221–238. 10.1016/j.jfda.2015.10.009 28911575 PMC9339571

[B34] Iriarte M. Murga N. Sagastagoitia D. Morillas M. Boveda J. Molinero E. (1993). Classification of hypertensive cardiomyopathy. Eur. Heart J. 14 (Suppl. J), 95–101. 8281972

[B35] Jiang B. Li D. Deng Y. Teng F. Chen J. Xue S. (2013). Salvianolic acid A, a novel matrix metalloproteinase-9 inhibitor, prevents cardiac remodeling in spontaneously hypertensive rats. PLoS One 8 (3), e59621. 10.1371/journal.pone.0059621 23533637 PMC3606118

[B36] Jin L. Wu D. (2006). The influence of modified Tianma Gouteng decoction on blood pressure heart function and cardiac ventricle remodeling in hypertensive heart disease. Hunan J. Tradit. Chin. Med. 22 (6), 3–5. 10.16808/j.cnki.issn1003-7705.2006.06.002

[B37] Ledwidge M. Gallagher J. Conlon C. Tallon E. O'Connell E. Dawkins I. (2013). Natriuretic peptide-based screening and collaborative care for heart failure: the STOP-HF randomized trial. JAMA 310 (1), 66–74. 10.1001/jama.2013.7588 23821090

[B38] Lee J. J. Yi H. Kim I. S. Kim Y. Nhiem N. X. Kim Y. H. (2012). (2S)-naringenin from Typha angustata inhibits vascular smooth muscle cell proliferation via a G0/G1 arrest. J. Ethnopharmacol. 139 (3), 873–878. 10.1016/j.jep.2011.12.038 22212500

[B39] Levy D. Larson M. G. Vasan R. S. Kannel W. B. Ho K. K. (1996). The progression from hypertension to congestive heart failure. JAMA 275 (20), 1557–1562. 10.1001/jama.1996.03530440037034 8622246

[B40] Li Y. Song P. Zhu Q. Yin Q. Y. Ji J. W. Li W. (2014). Liguzinediol improved the heart function and inhibited myocardial cell apoptosis in rats with heart failure. Acta. Pharmacol. Sin. 35 (10), 1257–1264. 10.1038/aps.2014.75 25220638 PMC4186991

[B41] Li N. Y. Yu H. Li X. L. Wang Q. Y. Zhang X. W. Ma R. X. (2018). Astragalus membranaceus improving asymptomatic left ventricular diastolic dysfunction in postmenopausal hypertensive women with metabolic syndrome: a prospective, open-labeled, randomized controlled trial. Chin. Med. J. (Engl.). 131 (5), 516–526. 10.4103/0366-6999.226077 29483384 PMC5850666

[B42] Li Q. Liu D. Chen M. (2019). Effect of Danxiong Tongluo Decoction combined with metoprolol on left ventricular diastolic function in patients with hypertensive heart disease. J. Sichuan Tradit. Chin. Med. 37 (09), 70–72.

[B43] Li M M. Han B. Zhao H. Xu C. Xu D. Sieniawska E. (2022). Biological active ingredients of Astragali Radix and its mechanisms in treating cardiovascular and cerebrovascular diseases. Phytomedicine 98, 153918. 10.1016/j.phymed.2021.153918 35104756

[B44] Li N N. Hang W. Shu H. Wen Z. Ceesay B. M. Zhou N. (2022). Salvianolic acid ameliorates pressure overload-induced cardiac endothelial dysfunction via activating HIF1[formula: see text]/HSF1/CD31 pathway. Am. J. Chin. Med. 50 (7), 1869–1885. 10.1142/S0192415X22500793 36121714

[B45] Lieu M. Koch W. J. (2019). GRK2 and GRK5 as therapeutic targets and their role in maladaptive and pathological cardiac hypertrophy. Expert. Opin. Ther. Targets 23 (3), 201–214. 10.1080/14728222.2019.1575363 30701991

[B46] Lin J. Wang Q. Zhou S. Xu S. Yao K. (2022). Tetramethylpyrazine: a review on its mechanisms and functions. Biomed. Pharmacother. 150, 113005. 10.1016/j.biopha.2022.113005 35483189

[B47] Liu C. (2012). Clinical study of 98 patients with hypertensive heart disease treated by combination of Chinese and Western medicine. China Health Care & Nutr 22 (16), 3514.

[B48] Liu C. Huang Y. (2016). Chinese herbal medicine on cardiovascular diseases and the mechanisms of action. Front. Pharmacol. 7, 469. 10.3389/fphar.2016.00469 27990122 PMC5130975

[B49] Liu D. Chen L. Zhao J. Cui K. (2018). Cardioprotection activity and mechanism of Astragalus polysaccharide *in vivo* and *in vitro* . Int. J. Biol. Macromol. 111, 947–952. 10.1016/j.ijbiomac.2018.01.048 29329811

[B50] Liu T. Zhang M. Niu H. Liu J. Ruilian M. Wang Y. (2019). Astragalus polysaccharide from Astragalus Melittin ameliorates inflammation via suppressing the activation of TLR-4/NF-κB p65 signal pathway and protects mice from CVB3-induced virus myocarditis. Int. J. Biol. Macromol. 126, 179–186. 10.1016/j.ijbiomac.2018.12.207 30586589

[B51] Liu M. Long X. Xu J. Chen M. Yang H. Guo X. (2022). Hypertensive heart disease and myocardial fibrosis: how traditional Chinese medicine can help addressing unmet therapeutical needs. Pharmacol. Res. 185, 106515. 10.1016/j.phrs.2022.106515 36265555

[B52] Lu X. R. Gao E. Xu L. Z. Li H. Z. Kang B. Chen W. N. (1987). Puerarin beta-adrenergic receptor blocking effect. Chin. Med. J. (Engl.). 100 (1), 25–28. 2885157

[B53] Lv S. Yuan P. Lu C. Dong J. Li M. Qu F. (2021). QiShenYiQi pill activates autophagy to attenuate reactive myocardial fibrosis via the PI3K/AKT/mTOR pathway. Aging (Albany NY) 13 (4), 5525–5538. 10.18632/aging.202482 33582656 PMC7950250

[B54] Ma L. Shao M. Cheng W. Jiang J. Chen X. Tan N. (2023). Neocryptotanshinone ameliorates insufficient energy production in heart failure by targeting retinoid X receptor alpha. Biomed. Pharmacother. 163, 114868. 10.1016/j.biopha.2023.114868 37201263

[B55] Mann D. L. Felker G. M. (2016). Heart failure: a companion to Braunwald’s heart disease. 3rd edition. Philadelphia: Elsevier-Saunders.

[B56] Messerli F. H. Rimoldi S. F. Bangalore S. (2017). The transition from hypertension to heart failure: contemporary update. JACC Heart Fail 5 (8), 543–551. 10.1016/j.jchf.2017.04.012 28711447

[B57] Mohammed S. A. D. Hanxing L. Fang L. Algradi A. M. Alradhi M. Safi M. (2023). Integrated Chinese herbal medicine with Western Medicine versus Western Medicine in the effectiveness of primary hypertension treatment: a systematic review and meta-analysis of randomized controlled trials. J. Ethnopharmacol. 300, 115703. 10.1016/j.jep.2022.115703 36096347

[B58] National Health Commission of the People’s Republic of China (2022). A list of items that are both food and medicine. Available online at: http://www.nhc.gov.cn/zwgk/wtwj/201304/3312183b2f954e35a29c77921a88d730.shtml (Accessed February 15, 2022).

[B59] National Health Commission of the People’s Republic of China (2023). Announcement on 9 new substances of Codonopsis pilosula and other new substances which are both food and traditional Chinese medicines in accordance with tradition. Available online at: http://www.nhc.gov.cn/sps/s7892/202311/f0d6ef3033b54333a882e3d009ff49bf.shtml (Accessed November 9, 2023).

[B60] Orgah J. O. He S. Wang Y. Jiang M. Wang Y. Orgah E. A. (2020). Pharmacological potential of the combination of Salvia miltiorrhiza (Danshen) and Carthamus tinctorius (Honghua) for diabetes mellitus and its cardiovascular complications. Pharmacol. Res. 153, 104654. 10.1016/j.phrs.2020.104654 31945473

[B61] Page M. J. McKenzie J. E. Bossuyt P. M. Boutron I. Hoffmann T. C. Mulrow C. D. (2021). The PRISMA 2020 statement: an updated guideline for reporting systematicreviews. BMJ 372, n71. 10.1136/bmj.n71 33782057 PMC8005924

[B62] Park W. H. Kim C. H. Lee Y. C. Kim C. H. (2004). Anti-inflammatory effects of a traditional Korean herbal formulation, Silsosangami, consisting of seven medicinal herbs: effect on hemolysis, neutrophil function, and gene expressions of iNOS and COX-2. Vasc. Pharmacol. 42 (1), 7–15. 10.1016/j.vph.2004.11.002 15664882

[B63] Peng X. (2015). Effects of Shexiang Baoxin Pill combined with amiodarone on heart rate, blood pressure and atrial fibrillation in hypertensive heart disease patients with atrial fibrillation in community. J. New Chin. Med. 47 (04), 16–17. 10.13457/j.cnki.jncm.2015.04.008

[B64] Qin F. Sun H. X. (2005). Immunosuppressive activity of Pollen Typhae ethanol extract on the immune responses in mice. J. Ethnopharmacol. 102 (3), 424–429. 10.1016/j.jep.2005.06.027 16095855

[B65] Ren W. Wang M. Liao J. Li L. Yang D. Yao R. (2020). The effect of Chinese herbal medicine combined with western medicine on vascular endothelial function in patients with hypertension: a systematic review and meta-analysis of randomized controlled trials. Front. Pharmacol. 11, 823. 10.3389/fphar.2020.00823 32612527 PMC7308496

[B66] Ritter O. Neyses L. (2003). The molecular basis of myocardial hypertrophy and heart failure. Trends Mol. Med. 9 (7), 313–321. 10.1016/s1471-4914(03)00114-x 12900219

[B67] Roth G. A. Johnson C. Abajobir A. Abd-Allah F. Abera S. F. Abyu G. (2017). Global, regional, and national burden of cardiovascular diseases for 10 causes, 1990 to 2015. J. Am. Coll. Cardiol. 70 (1), 1–25. 10.1016/j.jacc.2017.04.052 28527533 PMC5491406

[B68] Salmas R. E. Gulhan M. F. Durdagi S. Sahna E. Abdullah H. I. Selamoglu Z. (2017). Effects of propolis, caffeic acid phenethyl ester, and pollen on renal injury in hypertensive rat: an experimental and theoretical approach. Cell biochem. Funct. 35 (6), 304–314. 10.1002/cbf.3277 28833317

[B69] Selamoglu Talas Z. (2014). Propolis reduces oxidative stress in l-NAME-induced hypertension rats. Cell biochem. Funct. 32 (2), 150–154. 10.1002/cbf.2986 23788129

[B70] Slivnick J. Lampert B. C. (2019). Hypertension and heart failure. Heart fail. Clin. 15 (4), 531–541. 10.1016/j.hfc.2019.06.007 31472888

[B71] Song H. (2011). Observation of the effect of nourishing Yin and suppressing liver Yang decoction combined with western medicine on hypertensive cardiopathy. Mod. J. Integr. Tradit. Chin. West. Med. 20 (34), 4329–4330+4395.

[B72] Song B. (2019). The therapeutic effect of integrated Chinese and Western medicine in the treatment of hypertensive heart disease. Health friend 7 (97)–96.

[B73] Su X. Zhi X. Cui T. Zheng Q. Wang S. Cao Y. (2015). Vasorelaxant activities of Danhong injection and their differential effects on the rat abdominal aorta and mesenteric artery. J. Cardiovasc. Pharmacol. 65 (1), 62–71. 10.1097/FJC.0000000000000164 25264751

[B74] Su Q. Lv X. Ye Z. (2019). Ligustrazine attenuates myocardial injury induced by coronary microembolization in rats by activating the PI3K/akt pathway. Oxid. Med. Cell Longev. 2019, 6791457. 10.1155/2019/6791457 31191802 PMC6525935

[B75] Sun G. Li X. Wei J. Zhang T. Li B. Chen M. (2021). Pharmacodynamic substances in Salvia miltiorrhiza for prevention and treatment of hyperlipidemia and coronary heart disease based on lipidomics technology and network pharmacology analysis. Biomed. Pharmacother. 141, 111846. 10.1016/j.biopha.2021.111846 34225018

[B76] Tan Q. (2019). Curative effect of Chinese medicine combined with metoprolol on hypertensive heart disease. Inn. Mong. J. Tradit. Chin. Med. 38 (6), 43–44.

[B77] Tao R. (2021). Clinical effect of the method of phlegm-resolving and wind-calming therapy on patients with hypertensive heart disease. Qingdao Med. J. 53 (2), 148–150.

[B78] Tavolinejad H. Soltani D. Zargaran A. Rezaeizadeh H. Vasheghani-Farahani A. (2019). The story of amiodarone. Eur. Heart J. 40 (33), 2758–2759. 10.1093/eurheartj/ehz583 31505605

[B79] Tromp J. Paniagua S. M. A. Lau E. S. Allen N. B. Blaha M. J. Gansevoort R. T. (2021). Age dependent associations of risk factors with heart failure: pooled population based cohort study. BMJ 372, n461. 10.1136/bmj.n461 33758001 PMC7986583

[B80] Virani S. S. Alonso A. Benjamin E. J. Bittencourt M. S. Callaway C. W. Carson A. P. (2020). Heart disease and stroke statistics-2020 update: a report from the American heart association. Circulation 141 (9), e139–e596. 10.1161/CIR.0000000000000757 31992061

[B81] Wang J. Sun X. (2018). Clinical observation of modified Tongqiao Huoxue Decoction combined with Metoprolol tartrate tablet in the treatment of hypertensive heart disease. Hebei J. TCM. 40 (4), 566–568+627.

[B82] Wang Z. Wang J. (2012). Observation on the curative effect of Qi-yin nourishing therapy on hypertensive heart disease with left ventricular diastolic dysfunction. J. Emerg. Tradit. Chin. Med. 21 (7), 1145–1146.

[B83] Wang S. Niu T. Qi H. Geng Q. Wang J. Yang X. (2012). Clinical obsevation of therapy of traditional Chinese and western medicine on hypertensive heart disease. Shanxi J. TCM. 28 (5), 24–25.

[B84] Wang D. Fan G. Wang Y. Liu H. Wang B. Dong J. (2013). Vascular reactivity screen of Chinese medicine danhong injection identifies Danshensu as a NO-independent but PGI2-mediated relaxation factor. J. Cardiovasc. Pharmacol. 62 (5), 457–465. 10.1097/FJC.0b013e3182a29657 23921303

[B85] Wei Y. Wu Y. Feng K. Zhao Y. Tao R. Xu H. (2020). Astragaloside IV inhibits cardiac fibrosis via miR-135a-TRPM7-TGF-β/Smads pathway. J. Ethnopharmacol. 249, 112404. 10.1016/j.jep.2019.112404 31739105

[B86] WHO Traditional (2021). Complementary and integrative medicine. Available online at: https://www.who.int/health-topics/traditional-complementary-and-integrative-medicine#tab=tab_1 (Accessed May 22, 2021).

[B87] Wong K. H. Li G. Q. Li K. M. (2011). Kudzu root: traditional uses and potential medicinal benefits in diabetes and cardiovascular diseases. J. Ethnopharmacol. 134 (3), 584–607. 10.1016/j.jep.2011.02.001 21315814

[B88] Wright J. W. Mizutani S. Harding J. W. (2008). Pathways involved in the transition from hypertension to hypertrophy to heart failure. Treatment strategies. Treat. Strateg. Heart Fail. Rev. 13 (3), 367–375. 10.1007/s10741-007-9060-z 17987382

[B89] Xiong X. Yang X. Li X. Yue G. Xing Y. Cho W. C. (2019). Efficacy and safety of Chinese herbal medicine for patients with postmenopausal hypertension: a systematic review and meta-analysis. Pharmacol. Res. 141, 481–500. 10.1016/j.phrs.2019.01.018 30639372

[B90] Yang W. Zhou S. (2002). Clinical observation on effect of Danxiong Tongluo decoction in treating patients of hypertensive heart disease complicated with left ventricle diastolic malfunction. Chin. J. Integr. Tradit. West. Med. 22 (11), 819–821.

[B91] Yang B. Li H. Qiao Y. Zhou Q. Chen S. Yin D. (2019). Tetramethylpyrazine Attenuates the Endotheliotoxicity and the mitochondrial dysfunction by Doxorubicin via 14-3-3γ/Bcl-2. Oxid. Med. Cell Longev. 2019, 5820415. 10.1155/2019/5820415 31885804 PMC6914960

[B92] Yao Y. Lu Q. Hu Z. Yu Y. Chen Q. Wang Q. K. (2017). A non-canonical pathway regulates ER stress signaling and blocks ER stress-induced apoptosis and heart failure. Nat. Commun. 8 (1), 133. 10.1038/s41467-017-00171-w 28743963 PMC5527107

[B93] Ye X. Y. Song H. Lu C. Z. (2008). Effect of puerarin injection on the mRNA expressions of AT1 and ACE2 in spontaneous hypertension rats. Zhongguo Zhong Xi Yi Jie He Za Zhi. 28 (09), 824–827. 19065898

[B94] Yousefsani B. S. Jamshidi A. Dadmehr M. (2021). Herbal medicine in cardiovascular medicine: a discussion of herbal medications described by the Persian physician, Avicenna. Eur. Heart J. 42 (32), 3037–3039. 10.1093/eurheartj/ehab053 33638634

[B95] Yuan C. (2018). Analysis of the curative effect of integrated Chinese and Western medicine on hypertensive heart disease. Inn. Mong. J. Tradit. Chin. Med. 37, 41–42.

[B96] Zhang H. (2013). Clinical analysis of 68 cases of hypertensive heart disease treated by integrated Chinese and Western medicine. Mod. Diagn. Treat. 24 (13), 2923–2924.

[B97] Zhang G. Liu J. Gao M. Kong W. Zhao Q. Shi L. (2020). Tracing the edible and medicinal plant Pueraria Montana and its products in the Marketplace Yields subspecies level distinction using DNA Barcoding and DNA metabarcoding. Front. Pharmacol. 20, 336. 10.3389/fphar.2020.00336 32265708 PMC7098995

[B98] Zhang Q. Shao W. Xiao Y. Wang Y. Zhang J. Ao M. (2022). Chinese herbal medicine formula combined with calcium antagonist in the treatment of hypertension: a systematic review and meta-analysis. Clin. Exp. Hypertens. 44 (22), 181–190. 10.1080/10641963.2021.2013491 35000517

[B99] Zhao Q. (2022). Clinical analysis on the treatment of hypertensive heart disease by combining metoprolol tartrate Tablets with jiaweitongqiaohuoxue decoction. World J. Complex Med. 8 (12), 136–139.

[B100] Zhu Y. Fan M. Fan R. Yao W. Deng Z. Tian Z. (2019). Effect of Yifengyangtongluo prescription on the improvement of traditional Chinese medicine syndrome and heart function of hypertensive heart disease with Yang deficiency and collateral-blocking type. Chin. J. Tradit. Med. Sci. Technol. 26 (01), 54–55.

[B101] Zuo H. L. Linghu K. G. Wang Y. L. Liu K. M. Gao Y. Yu H. (2020). Interactions of antithrombotic herbal medicines with Western cardiovascular drugs. Pharmacol. Res. 159, 104963. 10.1016/j.phrs.2020.104963 32497719

