# Supplementary material for: Potential preventive effects of selected traditional Chinese medicine as adjuvant therapy on hypertensive heart disease progression by replenishing qi and activating blood circulation: a systematic review and meta-analysis of clinical trials
Source: Front Pharmacol. 2025 Oct 2;16:1506234. doi: 10.3389/fphar.2025.1506234 (PMC12528026; doi:10.3389/fphar.2025.1506234)
Supplement: Supplementary file 2 [file Table2.docx]

Supplementary Material 2

# Search strategies

| **Databases** | **Terms** |
| --- | --- |
| The Cochrane Library | #1 ("hypertensive heart disease" OR "hypertensive cardiovascular disease"):ti,ab,kw  #2 MeSH descriptor: [Medicine, Chinese Traditional] explode all trees  #3 ("Traditional Chinese Medicine" OR "Chung I Hsueh" OR "Hsueh, Chung I" OR "Traditional Medicine, Chinese" OR "Zhong Yi Xue" OR "Chinese Traditional Medicine" OR "Chinese Medicine, Traditional" OR "Drugs, Chinese Herbal" OR "Complementary Therapies" OR "Alternative Medicine"):ti,ab,kw  #4 #2 OR #3  #5 #1 AND #4 |
| PubMed | #1 (hypertensive heart disease[Title/Abstract]) OR (hypertensive cardiovascular disease[Title/Abstract])  #2 "Medicine, Chinese Traditional"[Mesh]  #3 (((((((((Traditional Chinese Medicine[Title/Abstract]) OR (Chung I Hsueh[Title/Abstract])) OR (Hsueh, Chung I[Title/Abstract])) OR (Traditional Medicine, Chinese[Title/Abstract])) OR (Zhong Yi Xue[Title/Abstract])) OR (Chinese Traditional Medicine[Title/Abstract])) OR (Chinese Medicine, Traditional[Title/Abstract])) OR (Drugs, Chinese Herbal[Title/Abstract])) OR (Complementary Therapies[Title/Abstract])) OR (Alternative Medicine[Title/Abstract])  #4 #2 OR #3  #5 #1 AND #4 |
| Embase | #1 'hypertensive heart disease'/exp  #2 'hypertensive cardiovascular disease':ab,ti  #3 'chinese medicine'/exp  #4 'medicine, chinese traditional':ab,ti OR 'traditional chinese medicine':ab,ti OR 'chung i hsueh':ab,ti OR 'hsueh, chung i':ab,ti OR 'traditional medicine, chinese':ab,ti OR 'zhong yi xue':ab,ti OR 'chinese traditional medicine':ab,ti OR 'chinese medicine, traditional':ab,ti OR 'drugs, chinese herbal':ab,ti OR 'complementary therapies':ab,ti OR 'alternative medicine':ab,ti  #5 #1 OR #2  #6 #3 OR #4  #7 #5 AND #6 |
| Web of Science | #1 (TS=(hypertensive heart disease)) OR TS=(hypertensive cardiovascular disease)  #2 TS=(Medicine, Chinese Traditional)  #3 (((((((((TS=(Traditional Chinese Medicine)) OR TS=(Chung I Hsueh)) OR TS=(Hsueh, Chung I)) OR TS=(Traditional Medicine, Chinese)) OR TS=(Zhong Yi Xue)) OR TS=(Chinese Traditional Medicine)) OR TS=(Chinese Medicine, Traditional)) OR TS=(Drugs, Chinese Herbal)) OR TS=(Complementary Therapies)) OR TS=(Alternative Medicine)  #4 #2 OR #3  #5 #1 AND #4 |
| China National Knowledge Infrastructure | (SU % '高血压性心脏病' OR SU % '高血压心脏病' OR SU % '高心病') AND (SU % '中医' OR SU % '中成药' OR SU % '中药' OR SU % '方药' OR SU % '中西医' OR SU % '传统医学' OR SU % '结合医学') |
| Wanfang Database | 题名:(高血压性心脏病 or 高血压心脏病 or 高心病) and 主题:(中医 or 中成药 or 中药 or 方药 or 中西医 or 传统医学 or 结合医学) |
| Chinese Biomedical Database | #1  "高血压性心脏病"[常用字段:智能] OR "高血压心脏病"[常用字段:智能] OR "高心病"[常用字段:智能]  #2 "医学, 中国传统"[不加权:扩展]  #3 "中医"[摘要:智能] OR "中成药"[摘要:智能] OR "中药"[摘要:智能] OR "方药"[摘要:智能] OR "中西医"[摘要:智能] OR "传统医学"[摘要:智能] OR "结合医学"[摘要:智能]  #4 #2 OR #3  #5 #1 AND #4 |
| Chinese Scientific Journal Database | 题名或关键词=(高血压性心脏病 or 高血压心脏病 or 高心病) AND 摘要=(中医 or 中成药 or 中药 or 方药 or 中西医 or 传统医学 or 结合医学) |

# indicates the step number.
